# Beneficial Effects of Exogenous Ketogenic Supplements on Aging Processes and Age-Related Neurodegenerative Diseases

**DOI:** 10.3390/nu13072197

**Published:** 2021-06-26

**Authors:** Zsolt Kovács, Brigitta Brunner, Csilla Ari

**Affiliations:** 1 Department of Biology, Savaria University Centre, ELTE Eötvös Loránd University, Károlyi Gáspár tér 4., 9700 Szombathely, Hungary; zskovacsneuro@gmail.com (Z.K.); brunnerb28@gmail.com (B.B.); 2 Faculty of Sciences, Institute of Biology, University of Pécs, Ifjúság Str. 6, 7624 Pécs, Hungary; 3 Behavioral Neuroscience Research Laboratory, Department of Psychology, University of South Florida, 4202 E. Fowler Ave, PCD 3127, Tampa, FL 33620, USA; 4 Ketone Technologies LLC, 2780 E. Fowler Ave. #226, Tampa, FL 33612, USA

**Keywords:** ketogenic supplement, ketosis, aging, lifespan, neurodegenerative disease, learning, memory

## Abstract

Life expectancy of humans has increased continuously up to the present days, but their health status (healthspan) was not enhanced by similar extent. To decrease enormous medical, economical and psychological burden that arise from this discrepancy, improvement of healthspan is needed that leads to delaying both aging processes and development of age-related diseases, thereby extending lifespan. Thus, development of new therapeutic tools to alleviate aging processes and related diseases and to increase life expectancy is a topic of increasing interest. It is widely accepted that ketosis (increased blood ketone body levels, e.g., β-hydroxybutyrate) can generate neuroprotective effects. Ketosis-evoked neuroprotective effects may lead to improvement in health status and delay both aging and the development of related diseases through improving mitochondrial function, antioxidant and anti-inflammatory effects, histone and non-histone acetylation, β-hydroxybutyrylation of histones, modulation of neurotransmitter systems and RNA functions. Administration of exogenous ketogenic supplements was proven to be an effective method to induce and maintain a healthy state of nutritional ketosis. Consequently, exogenous ketogenic supplements, such as ketone salts and ketone esters, may mitigate aging processes, delay the onset of age-associated diseases and extend lifespan through ketosis. The aim of this review is to summarize the main hallmarks of aging processes and certain signaling pathways in association with (putative) beneficial influences of exogenous ketogenic supplements-evoked ketosis on lifespan, aging processes, the most common age-related neurodegenerative diseases (Alzheimer’s disease, Parkinson’s disease and amyotrophic lateral sclerosis), as well as impaired learning and memory functions.

## 1. Introduction

Aging processes result in irreversible decline of normal physiological functions (time-dependent functional decline) and age-related diseases. It has been demonstrated that several genes and environmental factors can modulate cellular functions leading to the appearance of ageing hallmarks, such as cellular senescence, mitochondrial dysfunction, loss of proteostasis, telomere attrition, deregulated nutrient sensing, stem cell exhaustion and epigenetic alterations [1,2]. These changes may generate, for example, chronic inflammation and aging that leads to increased risk for age-related chronic diseases, such as neurodegenerative diseases (e.g., Alzheimer’s disease), osteoporosis, cardiovascular diseases, cancer, diabetes, sarcopenia and osteoarthritis [1,2].

A worldwide increase in elderly population has been predicted, as about 9% of people were over the age of 65 in 2019, which number was predicted to increase to approximately 17% by 2050 [3,4]. Human lifespan is increasing, as a result of more and more effective therapeutic tools and improvement in living conditions, but the health status of patients is not improving by the same intensity. Thus, the prevalence of age-related diseases, such as neurodegenerative diseases are continuously increasing each year [5,6] and the consequences of aging processes and related diseases generate enormous medical, psychological and economical burden for humanity [7]. To decrease the negative consequences of aging processes and related diseases, thereby to mitigate their negative effects on health and the economy, several drugs were developed that are undergoing clinical trials. For example, rapamycin and its analogues [8,9,10], metformin [11,12], sirtuin (SIRT) activators [13,14] and senolytics (for elimination of senescent cells) [15] can modulate aging mechanisms, and, as a consequence, increase lifespan and decrease risk for age-related diseases. However, to prevent, alleviate and delay age-related processes and diseases, to extend health span and to improve the quality of life of elderly population, development of safer and more effective drugs and therapeutic tools are needed.

Exogenous ketogenic supplements (EKSs), such as ketone esters (KEs, e.g., *R*,*S*-1,3-butanediol—acetoacetate diester), ketone salts (KSs, e.g., Na^+^/K^+^—β-hydroxybutyrate/βHB mineral salt), and medium chain triglycerides (MCTs/MCT oils containing, e.g., about 60% caprylic triglyceride and 40% capric triglyceride) have been proven effective when used together with normal diet to induce and maintain an increased blood ketone body level (ketosis) [16,17,18,19,20]. It has been demonstrated that the level of EKSs-induced ketosis may change by age and gender [21]. Ketone bodies (e.g., βHB and acetoacetate) can enter to the central nervous system (CNS) via monocarboxylate transporters and can be used for ATP (adenosine triphosphate) synthesis via the Krebs-cycle in brain cells [22,23,24,25]. It has been demonstrated that EKSs can generate rapid (0.5–6 h after administration) and mild to moderate [19,26,27,28,29] therapeutic ketosis (about 1–7 mM) [30,31]. In order to sustain therapeutic ketosis leading to positive outcome, administration of different amounts of EKSs must be repeated for several days or up to several months depending on the disease, the dose and type of EKSs. For example, administration of 30 g MCT drink/day for 6 months and 75 g KE/day for 4 weeks were able to evoke beneficial effects in patients with mild cognitive impairment and type 2 diabetes, respectively [32,33]. However, it has been suggested that not only these, but other EKSs may be effective and safe ketone body precursors for the treatment of diseases in humans through increased βHB level (ketosis) [29,32,34,35]. It has been demonstrated that EKSs are well-tolerated and safe (with mild adverse effects, if any) [19,26,28,29,33,36]. Moreover, administration of EKSs can circumvent both dietary restrictions and adverse effects of ketogenic diets (e.g., nephrolithiasis, constipation and hyperlipidemia) [37]. Thus, administration of EKSs may be a safe and effective alternative metabolic therapy to the ketogenic diet.

It has also been demonstrated that administration of EKSs-generated therapeutic ketosis may evoke beneficial effects on CNS diseases [34,38,39]. For example, KEs, KSs and MCT oils can evoke anti-seizure and anti-epileptic effects [36,40,41,42], anxiolytic influence [26,43,44], regeneration of nervous system injuries [45] and alleviating effects on neurodegenerative diseases (such as Alzheimer’s disease) [41,46,47,48]. These beneficial effects were induced likely through ketosis-evoked neuroprotective effects, for example, by improved mitochondrial functions, enhanced ATP levels, decreased inflammatory processes and decreased oxidative stress [23,24,34,49,50]. Moreover, ketone bodies may modulate aging processes thereby extend lifespan and delay the development of age-related diseases, such as neurodegenerative diseases. In fact, it has been demonstrated that not only ketogenic diets, but also administration of EKSs can increase and maintain blood ketone body level [19,26,27,28,29], which ketone bodies, such as βHB, may promote anti-aging effects [35,51,52]. Moreover, it was demonstrated that βHB, as an endogenous ligand molecule, can activate the hydroxycarboxylic acid receptor 2 (HCAR2 or GPR109A receptor) [53,54]. HCAR2 receptors are expressed not only in macrophages, but also in the brain cells, mainly in microglia, as well as astrocytes and neurons [54,55,56]. Thus, βHB molecule via, for example, HCAR2 receptors can modulate not only physiological, but also pathophysiological processes in the brain that are connected to aging and neurodegenerative diseases [55,57,58]. Based on the literature, increase of βHB level may be the main factor contributing to the beneficial effects on aging, lifespan and age-related diseases after administration of EKSs. Indeed, it has been demonstrated that βHB decreased the senescence associated secretory phenotype (SASP) of mammals [59] and extended the lifespan of *C. elegans* [60]. Consequently, in this review paper we focused on βHB-generated alleviating effects. Although limited evidence supports the alleviating influence of EKSs on lifespan, aging processes and related CNS diseases, we can hypothesize that EKSs-evoked increase in blood βHB level can modulate (alleviate) aging processes and improve symptoms of age-related diseases through their neuroprotective effects, therefore may delay both aging and the development of related diseases and extend lifespan.

This review discusses the hallmarks of aging and putative anti-aging molecular mechanisms (pathways) by which EKSs may be able to exert their beneficial effects on lifespan, healthspan, aging, the most common age-related neurodegenerative diseases (Alzheimer’s disease, Parkinson’s disease and amyotrophic lateral sclerosis), as well as learning and memory.

## 2. Main Features of Aging Processes

It has been demonstrated that aging is the most common risk factor for emergence of neurodegenerative diseases [2]. Indeed, as life expectancy of humans increase, more and more people suffer from different types of neurodegenerative diseases, such as Alzheimer’s disease [61]. Moreover, it has been demonstrated that development and incidence of the most common neurodegenerative diseases, Alzheimer’s disease (e.g., characterized by extracellular senile, amyloid-β/Aβ plaque and neurofibrillary tangle/hyperphosphorylated and misfolded Tau accumulation in the brain; impairment of learning and memory), Parkinson’s diseases (e.g., characterized by the accumulation of α-synuclein and the loss of dopaminergic neurons; tremors and muscle rigidity) and amyotrophic lateral sclerosis (e.g., accumulation of TAR DNA-binding protein 43; progressive degeneration of motor neurons a motor defects; muscle weakness) are promoted by aging [6,62,63,64]. It has also been also demonstrated that aging hallmarks, such as reduced telomere length and/or genomic instability, epigenetic alterations, mitochondrial dysfunction, cellular senescence, loss of proteostasis, changes in activity of nutrient sensing pathways and intercellular communication, as well as stem cell exhaustion can be detected in Alzheimer’s disease, Parkinson’s disease and amyotrophic lateral sclerosis. However, in amyotrophic lateral sclerosis, the reduced telomere length, genomic instability, cellular senescence and changes in intercellular communication may be the main contributing factors [63,64]. Thus, in this chapter, we shortly characterize the main aging hallmarks and their connection with the development of the above-mentioned age-related neurodegenerative diseases. Moreover, based on the literature (e.g., administration and effects of senomorphic drugs and caloric restriction) we present the main signaling pathways contributing to the modulation of aging processes, suggesting that inhibition or activation of these pathways may be used for delaying not only aging, but also related neurodegenerative diseases, improve impaired learning and memory functions, as well as to promote lifespan.

### 2.1. Nutrient Sensing Pathways

**Figure 1:**
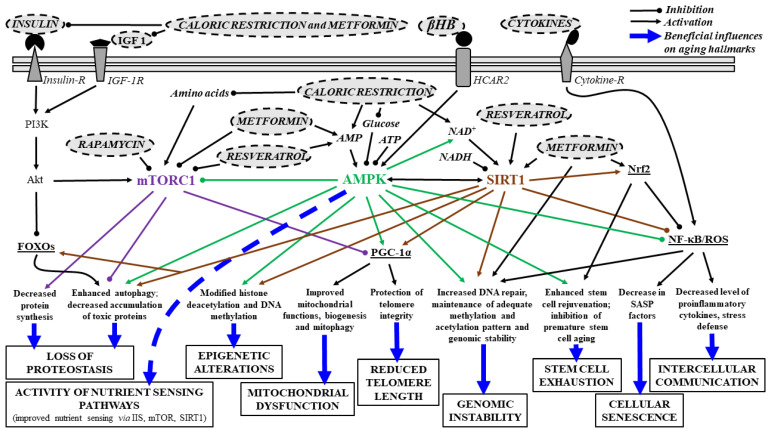
Main downstream signaling pathways and some effects, by which different senomorphic drugs (e.g., metformin), interventions (e.g., caloric restriction) and, theoretically, exogenous ketogenic supplements-evoked ketosis (βHB) can improve age-dependent impaired processes (aging hallmarks). Abbreviations: Akt, Akt kinase/protein kinase B; AMPK, AMP activated serine-threonine protein kinase; ATP, adenosine triphosphate; βHB, beta-hydroxybutyrate; FOXOs, Forkhead box Os; HCAR2, hydroxycarboxylic acid receptor 2; IGF 1, insulin-like growth factor 1; mTORC1, mechanistic target of rapamycin C1; NAD^+^, nicotinamide adenine dinucleotide; NADH, nicotinamide adenine dinucleotide (NAD) + hydrogen (H); NF-κB, nuclear factor kappa-light-chain-enhancer of activated B cells; Nrf2, nuclear factor erythroid 2-related factor 2; PGC-1α, peroxisome proliferator-activated receptor gamma (PPARγ) coactivator-1α; PI3K, phosphatidyl inositol-3-kinase; ROS, reactive oxygen species; SASP, senescence associated secretory phenotype; SIRT1, Sirtuin 1.

Changes in activity of nutrient sensing pathways may have a role in aging and development of age-related diseases. It has been demonstrated that caloric restriction and fasting can attenuate aging, expand lifespan, generate neuroprotective effects and prevent age-related diseases through energy (nutrient) sensing insulin/insulin-like growth factor (IGF) 1 (IIS) pathway, AMP (adenosine monophosphate) activated serine-threonine protein kinase (AMPK), Sirtuin 1 (SIRT1) and transcriptional factor FOXOs (Forkhead box Os) [65,66,67,68]. Previous studies show that caloric restriction can decrease IGF, insulin, glucose and amino acid levels, whereas increase NAD^+^ (nicotinamide adenine dinucleotide) and AMP levels (Figure 1). These alterations are sensed by the (i) IIS pathway, activated by increased IGF and glucose levels; (ii) AMPK, which senses low energy states via increased AMP levels; (iii) SIRT1, which also senses low energy states via increased NAD^+^ levels (NAD^+^-dependent protein deacetylase); and (iv) mechanistic target of rapamycin (mTOR), which senses high amino acid levels leading to stress resistance, oxidative metabolism, enhanced DNA repair, epigenetic stability and increase in longevity [69,70,71].

Reduced activity of the IIS pathways can extend lifespan [72], similarly to the mTOR inhibitor rapamycin-evoked increase in lifespan [9]. It was also demonstrated that decreased IIS signaling reduced the aggregation-mediated toxicity of the Aβ1–42 (amyloid β-peptide 1–42), suggesting that decreased insulin signaling may be protective against abnormal aggregation of proteins in neurodegenerative diseases, such as Alzheimer’s disease [73]. Moreover, mTOR (a serine/threonine protein kinase) is the main regulator of cellular growth and mass accumulation, which contains mTORC1 and mTORC2 complexes [6]. mTORC1 is able to integrate signals from nutrients, growth factors, energy, and oxygen level in order to promote cell proliferation and growth (e.g., enhancement of energy metabolism/glycolysis and nucleotide, protein, as well as lipid synthesis and inhibition of catabolism/autophagy) [74,75] (Figure 1). Indeed, for example, mTORC1 supports protein synthesis by phosphorylation of S6K1 (ribosomal protein S6 kinase 1) and 4EBP1 (eukaryotic translation initiation factor 4E binding protein 1) molecules, which processes may be activated by Akt kinase (protein kinase B) [6,75,76] (Figure 1). Moreover, mTORC1 can suppress autophagy via inhibition of ULK1 (Uncoordinated/Unc-51-like kinase 1) by which impedes the cellular homeostasis maintaining processes (e.g., providing nutrients under starvation and removing damaged organelles and misfolded proteins) [75,77]. Thus, inhibition of mTORC1 effects on autophagy may be an important tool to decrease age-dependent processes (aging hallmarks, such as loss of proteostasis) and promoting longevity [6] (Figure 1). It was also demonstrated that mTORC2 has a role in the cytoskeleton reorganization (connected to cell growth) and cell survival modulation [75,78].

SIRTs and AMPK also have a role in the modulation of lifespan. Activation of AMPK mediated pathways by low energy levels has a role in inhibition of glucose production, increase in activity of beta-oxidation (fat burning) and promotion of mitochondrial functions and mitochondrial biogenesis [79,80] (Figure 1). AMPK exerts its effect on energy metabolism by phosphorylation of, for example, (i) ACCs (acetyl-CoA carboxylases), such as ACC1, which ACC1 inhibition lead to enhancement of fatty acid oxidation/mitochondrial-oxidation and suppression of lipogenesis; and (ii) the transcription factor SREBP1 (sterol regulatory element-binding protein 1). The inhibitory effect of AMPK results in reduced fatty acid synthesis [80]. It was suggested that AMPK activation may be a promising anti-aging therapeutic target, for example, by improvement of mitochondrial dysfunction. AMPK activation not only decreased the activity of anabolic pathways and increased activity of catabolic pathways leading to increase of activity of energy (ATP)-generating pathways and decrease in energy (ATP)-consuming processes, but also increased lifespan in diabetic patients [79,80]. Moreover, increase in AMPK activity decreases the expression of proinflammatory cytokines, therefore modulate intercellular communication (Figure 1) by inhibition of advanced-glycation end products (AGEs)-evoked increase in the level of transcription factor NF-κB (nuclear factor kappa-light-chain-enhancer of activated B cells) mRNA and protein [81]. However, AMPK activation may suppress inflammation through the inflammatory response inducer NF-κB by other pathways, for example, through triggering of inhibitory activity of SIRT1, PGC-1α (peroxisome proliferator-activated receptor γ/PPARγ coactivator 1α), FOXOs and p53 (transcription factor tumor suppressor protein 53) on NF-κB-signaling or via inhibition of NF-κB activator ER (endoplasmic reticulum) stress and oxidative stress [82]. Moreover, AMPK is able to increase PGC-1α activity not only directly (by phosphorylation, before subsequent deacetylation of PGC1-α by SIRT1) [83], but also via arrest of PGC-1α inhibitory effect of mTORC1 [66] (Figure 1).

It has also been demonstrated that caloric restriction may exert its effect on lifespan through SIRTs [84], thus SIRTs are considered as putative anti-aging factors. SIRTs, such as SIRT1 and SIRT3 are able to sense low energy levels via detection of high NAD^+^ levels. SIRTs are Class III HDACs histone deacetylases, which enzymes use coenzyme NAD^+^ to remove acyl groups of proteins, such as acetyl-lysine residues of histones and non-histones, such as PGC-1α, FOXOs, p53 and NF-κB [69,85]. Under nutrient deprivation (caloric restriction), the level of a nutrient-sensing deacetylase SIRT1 is elevated (which, e.g., increases hepatic glucose production through PGC-1α), but its level reduced by overfeeding [86,87]. It has been demonstrated that activation (overexpression) of SIRT1 may increase lifespan and have an alleviating role in all age-related processes (hallmarks) (Figure 1) and several diseases, such as neurodegenerative diseases [88,89,90]. Indeed, SIRT1 expression was found to decrease with age, for example in the brain [91]. Moreover, it was also demonstrated that decreased level of SIRT1 in microglia can lead to cognitive decline (Tau-mediated memory deficits) in aging and neurodegeneration by upregulation of IL-1β (interleukin-1β) [91]. It was also demonstrated that caloric restriction can attenuate Alzheimer’s disease progression, for example, by decreasing the accumulation of Aβ plaque [92] and promote longevity and healthy aging [93] likely via SIRT1 activation [93,94,95], whereas higher caloric intake may increase the risk of the development of Alzheimer’s disease [96]. Reduction of SIRT1 levels was also demonstrated in parietal cortex in patients with Alzheimer’s disease, which was associated with the accumulation of Aβ and Tau [97], whereas activation of SIRT1 can suppress α-synuclein aggregation [98]. It has been demonstrated that SIRT1-evoked neuroprotection may evoke not only decrease in excitotoxicity and neurodegeneration [99,100], but also improved healthspan and extended lifespan likely through activation of PGC-1α (regulation of mitochondrial biogenesis) (Figure 1) and FOXOs (enhancing stress response via autophagy, resistance to oxidative stress and DNA damage and FOXO3′s ability to induce cell cycle arrest), as well as inhibition of p53 (regulation of apoptosis and cell cycle) and SREBP1 (regulation of lipid metabolism) activation [6,88,101,102]. These pathways can lead to alleviating effects in neurodegenerative diseases, such as Alzheimer’s disease and amyotrophic lateral sclerosis via, for example, SIRT1-generated deacetylation (and activation) of PGC-1α [94]. It has been demonstrated that SIRT1 is able to inhibit cell aging via p53 (deacetylation thereby inhibition of both p53 and its proapoptotic activity) [103] and can modulate development (fate) of neural progenitor cells [104]. It was also demonstrated that cellular NAD^+^ level decreased with age (evoked by, e.g., accumulated DNA damages during aging) leading to decreased SIRT activity, mitochondrial dysfunction [88,105] and development of age-related diseases, such as neurodegenerative diseases [106]. Consequently, therapeutic tools, such as administration of different drugs and metabolic therapies, which increase NAD^+^ level can evoke alleviating effects on aging-related processes and diseases, as well as promote longevity [6,106] (Figure 1).

It was also demonstrated that mutation, lacking, genetic variants or inactivation of insulin/IGF-1 receptor, as well as caloric restriction (inhibiting insulin/IGF-1 signaling) (Figure 1) extends the lifespan, not only in different animals, such as mice, but also in humans [6,107,108] via PI3K (phosphatidyl inositol-3-kinase)/Akt/FOXOs pathway promoting stress defense. Under these conditions (e.g., caloric restriction-evoked decrease in insulin level) unphosphorylated FOXOs can be transported to the nucleus to promote the transcription of several genes (namely, their phosphorylation impedes their translocation to the nucleus) leading to increased stress resistance, cell cycle arrest, damage repair and increased longevity (lifespan) [72,109].

### 2.2. Telomere Shortening and Genome Instability

Reduced length of repetitive ribonucleoprotein sequences at the distal ends of eukaryotic chromosomes (telomere) during cell division was demonstrated during physiological (“natural”) aging of mammals [110]. However, if the length of telomeres is too short it can cause damage of the DNA molecules, cellular senescence, mitochondrial dysfunctions (decreased mitochondrial biogenesis and functions, as well as increased ROS/reactive oxygen species level via p53-evoked repression of PGC-1α/β), and inflammation thereby aging [110,111,112]. It was also suggested that activation of telomerase activity not only enhances the survival time and increase lifespan of mammals [3,113], but also may be favorable for cancer cell development (by decreased senescence and immortalization) [2,114]. Thus, shorter telomeres- and low (if any) telomerase activity-evoked senescence can prevent tumorigenesis at least in animals with long lifespan [2]. It was also suggested that telomere attrition may have a role in development of age-related neurodegenerative diseases, such as Alzheimer’s disease [111]. AMPK and SIRT1 can attenuate age-related telomere shortening through PGC-1α (Figure 1) suggesting beneficial role of AMPK/SIRT1 activation on neurodegenerative diseases [115].

Not only telomere shortening, but also chromosomal aneuploidy, somatic mutations and copy mutations may have a role in DNA damage [116]. Moreover, defects of DNA repair mechanisms (such as base excision repair), mitochondrial DNA mutation and perturbations of the nuclear lamina may also generate genome instability (accumulation of genetic damage), cell dysfunction and aging via senescence [63,117,118,119], which processes may evoke (or have a role in) age-related diseases [78]. Indeed, DNA damage can trigger the onset of neurodegenerative diseases, such as Parkinson’s disease and amyotrophic lateral sclerosis [120]. Changes in integrity and stability of DNA can be evoked through both exogenous effects (e.g., by chemical, physical and biological agents) and endogenous influences (e.g., by increase in ROS level and DNA replication errors) [118]. SIRT1 have a positive influence on DNA repair thereby genomic instability (Figure 1), suggesting alleviating effect of SIRT1 activation on neurodegenerative diseases [115].

### 2.3. Epigenetic Alterations

The epigenome contains molecular switches by which genes may be activated or inhibited during the entire lifetime [121]. It was demonstrated that epigenetic alterations, such as changes in DNA methylation patterns (which methylation is inversely proportional to gene activation), chromatin remodeling, expression of non-coding RNAs and posttranslational histone modifications may also promote aging processes [78,122]. For example, it has been demonstrated that (hyper)methylation of promoter sequences of the genes (and in general on the DNA) can lead to silencing of genes related to, for example, apoptosis [123], whereas DNA hypomethylation promotes gene activation [124,125]. It was also demonstrated that changes in the pattern of DNA methylation (hypermethylation or hypomethylation) by age may be important in the mechanism of aging [126] and used as an aging clock (e.g., a link between methylcytosine/DNA methylation and age was demonstrated) [125,127]. Both global decrease of DNA methylation (which hypomethylation may induce age-associated genomic instability and loss of telomere integrity) and site-specific hypermethylation of promoter sequences were observed by age [122,123,124,128]. A previous study showed that age-induced hypomethylation was corrected by caloric restriction [129].

It has been suggested that caloric restriction can upregulate SIRT1 transcription leading to increase in histone deacetylation and methylation of DNA, which effects may compensate the decrease in both SIRT1 activity and DNA methylation, as well as increase in histone acetylation by age and increase lifespan (e.g., by maintenance of adequate DNA methylation pattern and genomic stability) [90,130] (Figure 1). Histone acetyl transferases (HATs) can attach acetyl groups to histones leading to increased positive charge, and attenuation of interaction with DNA, and thereby enhancing DNA transcription. Conversely, HDACs can remove acetyl groups from histones, which effect enhances interaction between histones and DNA resulting decreased transcription. Consequently, antagonists of HDACs may facilitate DNA transcription [131,132]. Based on these results above, expression of genes can be blocked (silenced) through not only methylation of DNA (e.g., methylation of promoter sequences of genes), but also deacetylation of histones, which continuous silencing of genes may be an important factor in progressive aging [123]. Moreover, histone methylation and demethylation (by histone methyl transferases and demethylases) and histone acetylation and deacetylation (by HATs and HDACs) can modulate lifespan, aging and age-related diseases [124,133,134]. For example, SIRT1-evoked deacetylation of Nk2 homeobox 1 can extend lifespan and delay aging processes in mice [133]. It has been demonstrated that inhibitors of HDACs (Classes I, II and IV HDACs), such as Trichostatin A, may be effective in the treatment of neurodegenerative diseases and the extension of lifespan [135,136]. Moreover, HDAC inhibitors decreased death of motor neurons, enhanced motor performance, increased the survival time and resulted in life extension in a mice model of amyotrophic lateral sclerosis [137], restored fear learning, decreased Aβ accumulation and improved cognitive performance in mouse models of Alzheimer’s disease [138,139] and generated neuroprotection in a model of Parkinson’s disease [140]. It was also suggested that miRNAs (microRNAs; a class of small non-coding silencing RNAs, which have a role in regulation of mRNA translation) may promote longevity and have a role in both neurodegeneration and age-related neurodegenerative diseases [141,142]. For example, hippocampal upregulation of miR-181 and related decrease of SIRT1 expression and, as a result, reduction of synaptic plasticity was demonstrated in a mouse model of Alzheimer’s disease [143]. As a response to severe, persistent DNA damage (e.g., by oxidative stress), activated poly(ADP-ribose)-polymerase-1 (PARP-1) adds ADP-ribose units to histones leading to the promotion of chromatin relaxation [144], enhances PARylation (generating PAR polymers as epigenetic effect) at sites of DNA damage (alteration) [63] and induce neuronal cell death via modulation of gene expression and mitochondrial dysfunction [145]. Moreover, excess PARP1 activation was demonstrated in aging and neurodegenerative diseases resulting mitochondrial dysfunction, neuroinflammation and dysregulation of autophagy (and mitophagy; e.g., via mTOR activation) [144,146]. For example, PARP1 enhances inflammation via NF-κB, decreased NAD^+^ level and SIRT1 activity and has a role in telomere shortening and, as a consequence, enhances senescence, leading to neurodegeneration and reduced lifespan [144,146,147]. As SIRT1 activity decreased by age [91], under this condition, both acetylation (activation) of PARP1, and PAPR1-evoked neuroinflammation may be increased. However, to retain its own functions via preservation of NAD^+^ levels, SIRT1 is able to deactivate (deacetylate) Parp1 [148]. Moreover, increased expression and excessive activation of PARP1 was demonstrated in Parkinson’s disease, Alzheimer’s disease and amyotrophic lateral sclerosis [145,149,150]. As it was demonstrated, Aβ and α-synuclein accumulation may generate activation of PAPR1 via, for example, increased level of ROS; thus, enhanced PARP1 activity aggravates Alzheimer’s disease and Parkinson’s disease symptoms by promotion of Aβ and α-synuclein aggregation, respectively [145,149]. Consequently, PARP1 inhibition can alleviate neuroinflammation, dysregulation of autophagy and mitochondrial dysfunction thereby inhibit development of inflammation(age)-related neurodegenerative diseases (or alleviate their symptoms), for example via SIRT1 activation [146,151]. It was also demonstrated that increase in βHB level can evoke epigenetic (posttranslational) gene regulation by β-hydroxybutyrylation of histones resulting regulation of gene expression thereby adaptation of cells to altered cellular energy source [152].

### 2.4. Mitochondrial Dysfunction

Mitochondrial dysfunction is associated with the decline of mitochondrial activity, such as defect of respiratory chain, decrease in ATP synthesis and level, as well as increase in ROS production. This hallmark of aging may be evoked by, for example, decreased mitochondrial biogenesis, defective mitophagy and mtDNA mutations leading to processes (e.g., enhancement of inflammatory processes), which can reduce lifespan, enhance aging and the risk of age-related diseases [69,153]. Indeed, it has been demonstrated that decrease in mitochondrial functions or damage of mitochondria may also be in the background of the development of neurodegenerative diseases [154] through excessive ROS formation leading to inflammation and genomic instability. These processes can enhance cellular senescence, aging processes and development of age-related diseases [154]. It was also demonstrated, that increased level of ROS may generate protective, homeostatic (alleviating) processes (e.g., on lifespan limiting cellular processes via ROS-dependent, protective, stress-response pathways), but, by aging progress, above a certain level ROS can evoke (aggravate) age-related damages [155]. It was demonstrated that autophagy (and mitophagy) declined with age [156], which can generate accumulation of damaged mitochondria thereby increased inflammation (e.g., via increased ROS level-evoked activation of NLRP3/NOD-like receptor pyrin domain 3 and NF-κB), cell death (e.g., through activation of caspases and mitochondrial permeability transition/mPT pore by excess ROS) and DNA damage (by ROS leading to increase in apoptotic signaling, such as p53) [153]. Moreover, it has been demonstrated that defects in mitochondria and autophagy (thereby aggregation of not only α-synuclein and Aβ peptide, but also impaired mitochondria) may have a role in development of neurodegenerative diseases, such as Parkinson’s disease and Alzheimer’s disease [153,156,157,158]. Thus, drugs or interventions, such as caloric restriction, which are able to promote autophagy and mitophagy, therefore inhibit mitochondrial dysfunction, ROS production, aggregation of toxic proteins, inflammation, cell death and cell senescence, can delay age-related degeneration, extend healthy lifespan and alleviate neurodegenerative diseases [159,160,161]. Indeed, for example, it was demonstrated that SIRT1 has a role in elimination of damaged mitochondria via autophagy (by enhanced activity of autophagy proteins) [162,163,164] and in mitochondrial biogenesis (increase in mitochondrial biogenesis) via increased transcriptional cofactor PGC-1α activity [87] (Figure 1), whereas a mitochondrial deacetylase SIRT3 controls (decreases) ROS level by enhancement of antioxidant activity of superoxide dismutase 2 (SOD2) during caloric restriction, leading to increased oxidative stress resistance [165]. Moreover, it was also demonstrated that increased SIRT3 activity can suppress mPT pore formation by which it can prevent mitochondrial dysfunctions [166]. It was also demonstrated that PGC-1α activation can enhance mitochondrial biogenesis and improve mitochondrial energy metabolism, therefore increasing lifespan and protecting against neurodegenerative diseases [167]. PGC-1α can bind and co-activate the transcription factor PPARγ (belongs to the superfamily of nuclear receptors) and promotes not only mitochondrial biogenesis, but also SOD and catalase activity, glucose metabolism and oxidative phosphorylation [162,168,169,170], whereas reduces the level of NF-κB and pro-inflammatory cytokines [171,172], as well as Aβ generation [173,174]. Indeed, reduced level of PGC1-α can result in decreased mitochondrial respiration and enhanced inflammatory processes [175]. Moreover, mitochondrial uncoupling via the overexpression of uncoupling protein 1 (UCP1) may also increase the lifespan [176].

### 2.5. Altered Intercellular Communication: Increased Inflammatory Processes

Aging processes are also connected to dysregulation of cell-cell connectivity and intercellular communication leading to, among others, sterile (activation of immune response without appearance of pathogens), chronic, low-grade inflammation (named “inflammaging”) with activation of NF-κB, as well as increased synthesis and release of proinflammatory cytokines (e.g., IL-1β and TNF-α/tumor necrosis factor-α) [69,125,177,178]. Increase in inflammatory processes and proinflammatory cytokine levels can also enhance (trigger) aging processes, for example, through increased activation of intracellular multiprotein sensor NLRP3 inflammasome, senescent cells-evoked release of proinflammatory cytokines and NF-κB level and signaling [177,179,180]. Autophagy failure in old organisms (e.g., decrease in activity of autophagy), and in patients with Alzheimer’s disease and Parkinson’s disease [181,182] were also demonstrated. It was suggested that aging (e.g., decreased autophagy by age) can stimulate NF-κB signaling, which transcription factor NF-κB (similar to increase in ROS by mitochondria and aggregation of Aβ) stimulate inflammatory processes, for example, via increased NLRP3 expression and IL-1β release [161,179,183,184,185], whereas autophagic uptake of damaged mitochondria (resulting decrease in ROS level) suppresses NLRP3 stimulation [161]. Thus, it was suggested that autophagy may generate an anti-inflammatory effect by inhibition of NLRP3 inflammasome thereby mitigating the NLRP3-evoked cleavage of pro-IL-1β to its active form/IL-1β by caspase-1 [186,187] leading to delay in aging processes [180].

Moreover, responsiveness of AMPK signaling decreased by age [180,188], which mitigates its inhibitory activity on NF-κB signaling [82] (Figure 1) and impairs autophagic activity leading to increased oxidative stress and activation of inflammasomes [180] and can attenuate lifespan [82]. As mTORC1 is able to inhibit autophagy (e.g., mitophagy or macroautophagy of altered proteins) all of drugs or interventions, which are able to inhibit mTORC1 (e.g., caloric restriction leading to mTOR inhibition) may be potent delayer of aging processes and enhancer of lifespan via inhibition of inflammation [180] (Figure 1), by which can alleviate not only neuroinflammation, but also neurodegeneration and related diseases, such as Alzheimer’s disease, Parkinson’s disease and amyotrophic lateral sclerosis [183,189]. Indeed, inhibition of NF-κB signaling was able to prevent age-associated features in mouse models extending their longevity [190].

### 2.6. Cellular Senescence

Cellular senescence can be evoked by intracellular and extracellular, genomic and epigenomic harmful stimuli and damages resulting hallmarks of aging (e.g., age-related stress: oxidative stress and telomere shortening; metabolic, as well as ER stress; mitochondrial dysfunction, loss of proteostasis) [191,192,193]. One of the main features of aging is the enhancement of cellular senescence (irreversible cell-cycle arrest regulated by, e.g., telomere attrition/DNA damage-evoked p53-dependent DNA-damage response, in which p53 is activated). Excessive accumulation of senescent cells, which cells decrease tissue regeneration and resistant to apoptosis (e.g., by upregulation of antiapoptotic Bcl-2/B cell lymphoma-2 family proteins resulting resistance to apoptosis-inducing signals), can evoke harmful processes on surrounding cells by secretion of proinflammatory agents (SASP factors, e.g., IL-1β,) and other components (e.g., IGF-1) [2,191,194,195]. For example, previous studies show that acute administration of IGF-1 can promote cell proliferation and survival whereas prolonged administration of IGF-1 promotes cell growth arrest and senescence (and the latter, enhances aging processes and inhibits tumorigenesis) via through SIRT1 inhibition and increased p53 activity (by increased acetylation) [196] and suppression of autophagy (e.g., via mTOR) [197] (Figure 1). Indeed, SIRT1 can inhibit not only DNA-damage, but also cellular senescence via deacetylation (inhibition) of p53 resulting anti-aging effects [198]. In contrast with cellular senescence, cellular quiescence occurs when nutrition or growth factor levels are very low (or lack) leading to a reversible cell-cycle arrest. In this state the cells may impede initiation of cell senescence [199] and has a role in maintenance of stemness [200]. However, in relation to maintaining cellular balance, senescence of cells is a double-edged sword [2]. For example, cellular senescence can reduce liver fibrosis [201], promote tissue repair and has a role in not only physiological, but also pathophysiological processes (e.g., embryogenesis and wound healing) [195] and prevent cancer development [202], but exaggerated attenuation of processes of cell senescence and accumulation of senescent cells can generate (or enhance) aging, and, as a consequence, development of age related diseases, such as Alzheimer’s disease and cancer [192,195,203,204,205]. Thus, medication of cellular senescence needs careful attention. Under glucose deprived condition, AMPK-induced p53 activation potentiates cellular survival (p53-dependent metabolic arrest), but excessive (lasting) AMPK activation leads to enhanced p53-dependent cellular senescence [206,207]. However, not only SIRT1, but also AMPK activation can improve cellular senescence via, for example, inhibition of proinflammatory mediators [5,81,82] (Figure 1).

### 2.7. Loss of Proteostasis and Stem Cell Exhaustion

Impaired protein homeostasis (loss of proteostasis) by age may also be in the background of aging and related diseases (e.g., neurodegenerative diseases) leading to dysregulation of protein synthesis, degradation and protein aggregation, disaggregation, assembly, folding and trafficking [208]. For example, activity of ubiquitin-proteasome system and autophagy decreased with age [209], whereas increased activity of proteostasis network (e.g., enhanced autophagy) extended the healthspan and lifespan [210]. Inhibition of mTOR pathways (e.g., by caloric restriction through decreased protein synthesis and activation of autophagy) may improve protein homeostasis and extend lifespan [211,212] (Figure 1). It has been demonstrated that maintained mitochondrial proteostasis prolonged lifespan and reduced Aβ protein aggregation in Alzheimer’s disease models [213]. Moreover, decreased activity of autophagy-lysosomal pathway may have a role in the development of both Alzheimer’s disease and Parkinson’s disease and other neurodegenerative diseases [77]. Indeed, activation of mitophagy (by which autophagy-lysosomal pathway remove damaged/dysfunctional mitochondria) was able to increase lifespan in worms and reverse cognitive deficits in models of Alzheimer’s disease [214,215]. AMPK activation may participate in maintenance of proteostasis via inhibition of mTOR and phosphorylation of eIF2α (eukaryotic initiation factor 2α; resulting attenuation of protein synthesis) and via activation of autophagy [79,80] (Figure 1). Moreover, it was also demonstrated that autophagy may be enhanced via inhibition of mTOR by SIRT1 [216] (Figure 1). Thus, activation of AMPK/SIRT1 and inhibition of mTOR (mTORC1, but not mTORC2 because the latter is required for autophagy) activity may be a promising target in anti-aging therapy [77]. Indeed, aging and age-associated diseases can upregulate mTORC1 [69].

Stem cell exhaustion may have a role in aging and appearance of age-related diseases through loss of regenerative ability of cells, tissues and organs. For example, activity and number of hematopoietic cells and intestinal stem cells are decreased by age leading to decrease in lymphoid cell number and adaptive immune response, increase in risk of anemia development and myeloid cell number, as well as malfunctions in intestinal functions [217,218]. Moreover, age-dependent decrease in function of other stem cells, such as neuronal stem cells was also demonstrated [71]. It was suggested that stem cell aging may be evoked by several factors, such as DNA damage and mutation, cellular senescence, defects in proteostasis, mitochondrial dysfunction and telomere attrition [63,71].

Thus, we can conclude that activation of AMPK/SIRTs-modulated signaling pathways, inhibition of mTOR effects (e.g., by inhibition of IIS pathway) and modulation of gene expression (e.g., by HDAC inhibitors) can alleviate aging processes (hallmarks) through direct and indirect manner (e.g., improvement of one of aging hallmarks, such as telomere attrition can improve other aging hallmarks, such as senescence and mitochondrial dysfunction), leading to extended lifespan and delay the appearance of neurodegenerative diseases.

### 2.8. Effects of Senotherapeutic Drugs on Aging Hallmarks and Neurodegenerative Diseases: Main Signaling Pathways

It has been demonstrated that elimination of senescent cells by senolytics (such as senolytic cocktails containing quercetin and dasatinib) can evoke alleviating effects on age-related diseases, such as Alzheimer’s disease and Parkinson’s disease and improve healthspan in aged humans [192,203,219]. Another senotherapeutic strategy is the administration of senomorphics (e.g., metformin and rapamycin) to alleviate (abolish) features of senescence (e.g., decrease in production and release of SASP factors) (Figure 1) without elimination of senescent cells, which may delay both aging and development of age-related diseases [194]. It was suggested that mTOR has a role in, among others, lifespan control [220]. Indeed, rapamycin (sirolimus; as an mTOR inhibitor) (Figure 1) is able to decrease the risk of development of age-related diseases, such as neurodegenerative diseases, to improve age-related decrease in memory and learning functions and to extend longevity [74,220]. Rapamycin decreased accumulation of Aβ and Tau leading to decreased loss of neurons, attenuated neuroinflammation and alleviated cognitive dysfunction in mouse models of Alzheimer’s disease [221]. Resveratrol also promotes clearance of Aβ peptides [95], likely via inhibition of mTOR and activation of AMPK [5] and prevents cognitive impairment [222] in different cell lines and models of Alzheimer’s disease. Thus, these results suggest that resveratrol and rapamycin exert its neuroprotective, alleviating effect on health span, lifespan and age-associated diseases likely by modulation of autophagy and proteostasis (via mTOR inhibition), as well as inflammation, among others [211,212,220] (Figure 1). Rapamycin and metformin (an antidiabetic drug, which reduces IGF levels, insulin resistance and insulin level) reduced accumulation of α-synuclein and improved behavioral impairments in models of Parkinson’s disease [74,220,223]. Moreover, metformin inhibits the mitochondrial electron transport chain (ETC complex I: NADH/ubiquinone oxidoreductase; thereby oxidative phosphorylation), consequently, cytoplasmic AMP/ATP and ADP/ATP ratios were increased resulting direct activation (phosphorylation) of AMPK [224,225] and decrease in ROS level [226]. Activation of AMPK (e.g., by metformin) (Figure 1) enhances mitochondrial biogenesis (via SIRT1/PGC-1α) and lipid beta-oxidation (via ACCs), inhibits hepatic glucose production and alleviates proteostasis (via mTOR inhibition), enhances autophagy (via mTOR inhibition and activation of ULK1), evokes hypoglycemia (decreasing plasma glucose levels, e.g., via improved hepatic insulin sensitivity leading to decrease in hepatic glucose production), improves nutrient sensing (via IIS/mTOR/SIRT1 pathways), inhibits NF-κB, improves DNA repair and decreases the level of proinflammatory cytokines (e.g., via activation of SIRT1) [6,12,225,226,227,228] leading to alleviating effects on aging-processes and related neurodegenerative diseases. As AMPK-independent influences, metformin is able to inhibit ROS production (via, e.g., inhibition of mitochondrial ETC and activation of antioxidant transcription factor nuclear factor erythroid 2-related factor 2/Nrf2) (Figure 1), enhance autophagy (through direct inhibition of mTOR), enhance SIRT1 activity (especially when NAD^+^ level highly reduced), activate DNA-damage-like response (and facilitates DNA repair likely via p53), attenuate NF-κB signaling and synthesis (release) of proinflammatory cytokines, inhibit SASP factors via Nrf2 and decrease level of insulin and IGF-1 levels thereby insulin/IGF-1 signaling (by which decreases mTOR activity) [66,86,225,229,230,231,232]. All of these processes can increase lifespan and evoke alleviating effects on both ageing and age-related diseases, such as Alzheimer’s disease and Parkinson’s disease [224,225,233]. Moreover, metformin is able to inhibit premature stem cell aging (via Nrf2), enhance stem cell rejuvenation (through AMPK) [234], affect histone modifications (e.g., via activation of SIRT1, inhibition of Class II HDACs and HAT phosphorylation) through AMPK-dependent and independent pathways [235], increase the levels of several miRNAs, which are implicated in the regulation of aging and cellular senescence, likely via AMPK [236] and reduce telomere shortening (e.g., via AMKP/PGC-1α/telomeric repeat-containing RNA/TERRA pathway; TERRA is transcribed from telomeres and has an important role in protection of telomere integrity) [225,237,238] (Figure 1). Indeed, it was demonstrated that, activation of AMPK can both enhance gene expression (e.g., by phosphorylation/inactivation of HDACs and activation of HAT1-evoked acetylation of histones) and inhibit gene transcription (e.g., via enhanced cellular NAD^+^ levels, and, as a consequence, increased SIRT1 deacetylation activity) [235]. Moreover, resveratrol can also extend lifespan and prevent neurodegenerative diseases [239]. For example, resveratrol can generate anti-inflammatory and anti-oxidative effects (e.g., decreases level of ROS, p53, NF-κB and proinflammatory cytokines, such as TNF-α and IL-1β) [5] and increase mean life expectancy and maximal life span in models of Alzheimer’s disease [240]. Moreover, resveratrol improved motor neuron function and extended the lifespan in a mouse model of amyotrophic lateral sclerosis [241]. It was suggested that resveratrol may exert its effects via activation of AMPK/SIRT1-modulated pathways [242] (Figure 1) by which this drug can deacetylate several substrates, such as p53, PGC-1α, FOXOs (e.g., FOXO3) and SREBP1 leading to induction of cell cycle arrest, mitochondrial biogenesis, DNA repair, oxidative stress response, autophagy and regulation of lipid metabolism [6,101,243]. For example, SIRT1 can decrease ROS and NF-κB-evoked effects (e.g., neuroinflammation) via Nrf2 [5] (Figure 1). However, it was also suggested that not only SIRT1, but also PGC1-α can increase the expression of Nrf2 [244,245] and AMPK enhances the nuclear translocation of the Nrf2 [246]. Based on these results, more effective sirtuin-activating compounds were developed, such as SRT2104, which drug may be a promising anti-aging drug (e.g., it increased lifespan and decreased inflammatory processes) [247]. Other natural products, such as curcumin, berberine and quercetin [6] can also generate positive effects on lifespan (by slowing aging), age and age-related diseases, for example, through AMPK activation and mTOR inhibition (e.g., to induce autophagy), activation of SIRT1 (to promote mitochondrial biogenesis) and anti-inflammatory effects [74,248,249,250].

Thus, administration of senotherapeutic drugs suggests that therapeutic tools and drugs, which are able to modify aging processes through activation or inhibition of certain signaling pathways can also delay development (or improve symptoms) of neurodegenerative diseases (such as Alzheimer’s disease, Parkinson’s disease and amyotrophic lateral sclerosis), improve memory and learning functions, as well as extend longevity.

## 3. Alleviating Effects of Ketosis on Lifespan, Aging and Age-Related Neurodegenerative Diseases

### 3.1. Ketosis-Evoked Neuroprotective Effects and Downstream Signaling Pathways

It has been demonstrated that ketosis and administration of βHB (as an alternative energy fuel to glucose) can increase mitochondrial ATP production and ATP release leading to increased extracellular level of purine nucleoside adenosine (via metabolism of ATP) [251,252,253]. Adenosine can activate its receptors leading to reduced oxidative stress (ROS level) [254] and reduce inflammatory processes [255]. Indeed, as enhanced level of ROS may activate (open) mPT pore thereby uncouple electron transport system from ATP production, βHB-evoked decrease in ROS production [94] can improve mitochondrial respiration and ATP production [49]. It was also suggested that therapeutic ketosis can increase the inhibitory GABAergic effects [22,256], decrease glutamate release and glutamate-induced neuronal excitability [256,257] and modulate (increase) the level of dopamine, adrenaline, noradrenaline and serotonin [258,259].

As an epigenetic gene regulator, βHB can inhibit the activity of the classical HDAC family (Class I and Class IIa HDACs) leading to enhanced acetylation of histone residues, thereby DNA can be accessed for transcription factors, such as FOXO3A [53,132,260]. FOXO3A generates enhanced expression of various antioxidants genes, enhances mitochondrial homeostasis (e.g., by regulation of mitochondrial biogenesis and ATP synthesis) and decreases oxidative stress [260,261]. Moreover, decrease in oxidative stress can also be generated by βHB-evoked inhibition of HDACs via attenuation of ER stress [262]. It has also been demonstrated, that the expression of brain-derived neurotrophic factor (BDNF) may be increased through βHB-evoked inhibition of HDACs [263] by which βHB evokes anti-inflammatory effects (via inhibition of both NLRP3, NF-κB and proinflammatory cytokine levels) [264,265], increases mitochondrial respiration and ATP levels [266], enhances the activity of anti-oxidant enzymes (such as SOD), and protects tissues against glutamate-induced excitotoxicity [267,268]. It has been also demonstrated that βHB can modulate gene expression through promotion of histone and non-histone acetylation by HATs [266,269]. Moreover, βHB is able to directly bind to an RNA-binding protein hnRNP A1 (heterogeneous nuclear ribonucleoprotein A1), which protein regulates, for example, RNA processing and function, as well as stabilization of mRNA [59,270,271].

Previous studies showed that βHB, through HCAR2, activates AMPK leading to NAD^+^-generation, which increases activity of SIRTs (e.g., SIRT1 and SIRT3; βHB/HCAR2/NAD^+^/SIRTs pathways) [272] (Figure 1) and thereby evoke neuroprotective effects [53,83,273,274]. Through both βHB/HCAR2/AMPK/SIRT1/NF-κB pathway and βHB/HCAR2/AMPK/mTOR pathway, βHB may generate anti-inflammatory effects by, for example, inhibition of proinflammatory transcription factor NF-κB and enhancement of autophagy, respectively [55,272,275], leading to decreased level of proinflammatory agents (e.g., TNF-α, IL-1β) [50,55,57,276]. βHB/HCAR2/AMPK/SIRT1/FOXO3A pathway can evoke antioxidant influences, thereby decrease in oxidative stress by increased expression of genes of the antioxidants (e.g., manganese superoxide dismutase/MnSOD: βHB/HCAR2/AMPK/SIRT1/FOXO3A/MnSOD pathway) [164,277]. Ketone bodies increase the expression of not only HCAR2 [278,279], but also SIRTs (e.g., SIRT1 and SIRT3) and PGC1-α [164,278,280]. These results suggest that both βHB/HCAR2/AMPK/SIRT1/PGC1-α and βHB/HCAR2/AMPK/SIRT3/PGC1-α pathways can function in the CNS. Indeed, neuroprotective influences of PGC1-α (e.g., anti-inflammatory effects and promotion of mitochondrial functions) can be modulated through not only SIRT1, but also SIRT3 [278,281,282,283]. It was also suggested that βHB-evoked effects on mitochondrial functions (e.g., mitochondrial biogenesis) may be generated through βHB/HDAC/BDNF/PGC1-α pathway [284]. Moreover, ketosis can enhance the expression of PPARs and the activity of the Nrf2 in the brain likely through βHB/HCAR2/AMPK/Nrf2 or βHB/HCAR2/AMPK/SIRTs/PGC1-α/Nrf2 pathway [285,286,287]. It has been suggested that ketosis may enhance expression of UCPs, therefore decrease the production of ROS [23,288,289] and defend mitochondria and mitochondrial functions (e.g., by reduction of oxidative stress) through activation of βHB/HCAR2/AMPK/SIRT3/PGC1-α/UCP1 pathway [283] and/or βHB/HCAR2/AMPK/SIRT3/PGC1-α/UCP2 pathway [278]. Moreover, not only ketosis (βHB), but also decrease in glucose level can mitigate inflammatory processes through decreased NLRP3 inflammasome activity. Namely, βHB is an endogenous inhibitor of NLRP3 inflammasome, likely via βHB/NLRP3/IL-1R (IL-1 receptor)/NF-κB pathway, whereas increased glucose level may enhance activity of NLRP3 and inflammatory processes. In addition, enhanced glucose level generally increases insulin level leading to decrease in ketone body synthesis [290,291,292]. EKSs were proven to decrease glucose levels [21,26,28,36,293], thereby they may increase activity of AMPK/SIRTs signaling pathways and inhibit mTOR-evoked effects (Figure 1).

**Figure 2:**
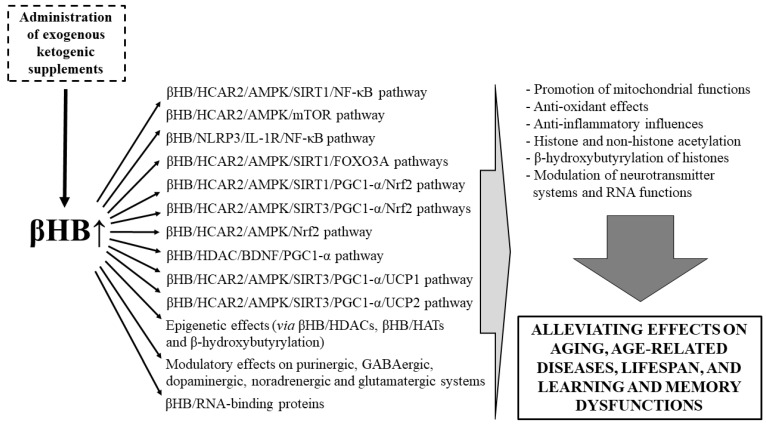
Signaling pathways and effects by which exogenous ketogenic supplements-generated ketosis (βHB) may extend lifespan, delay both aging and development of neurodegenerative diseases, and improve learning and memory dysfunctions. Abbreviations: AMPK, AMP activated serine-threonine protein kinase; βHB, beta-hydroxybutyrate; BDNF, brain-derived neurotrophic factor; FOXO, Forkhead box O; HATs, histone acetyltransferases; HCAR2, hydroxycarboxylic acid receptor 2; HDAC, histone deacetylase; IL-1R, IL-1 receptor; mTOR, mechanistic target of rapamycin; NF-κB, nuclear factor kappa-light-chain-enhancer of activated B cells; NLRP3, NOD-like receptor pyrin domain 3; Nrf2, nuclear factor erythroid 2-related factor 2; PGC1-α, peroxisome proliferator-activated receptor gamma (PPARγ) coactivator-1α; ROS, reactive oxygen species; SIRT, sirtuin; UCP, uncoupling protein.

Thus, based on previous studies, βHB/HCAR2/AMPK/SIRT1/NF-κB, βHB/HCAR2/AMPK/mTOR and βHB/NLRP3/IL-1R/NF-κB pathways (anti-inflammatory effects), βHB/HCAR2/AMPK/SIRT1/FOXO3A pathway (improving mitochondrial functions, anti-oxidant influences), βHB/HCAR2/AMPK/SIRT1/PGC1-α/Nrf2, HCAR2/AMPK/SIRT3/PGC1-α/Nrf2 and HCAR2/AMPK/Nrf2 pathways (improving mitochondrial functions, anti-oxidant and anti-inflammatory effects), βHB/HDAC/BDNF/PGC1-α pathway (improving mitochondrial functions; anti-oxidant and anti-inflammatory influences), βHB/HCAR2/AMPK/SIRT3/PGC1-α/UCP1 and/or βHB/HCAR2/AMPK/SIRT3/PGC1-α/UCP2 pathways (anti-oxidant and anti-inflammatory effects, improving mitochondrial functions) and modulatory effects of βHB on neurotransmission (e.g., purinergic, GABAergic, dopaminergic, noradrenergic and glutamatergic systems), gene expression (e.g., enhanced acetylation of histone residues via βHB/HDACs, promotion of histone and non-histone acetylation through βHB/HATs and hydroxybutyrylation of histones) and RNA functions (e.g., via RNA-biding proteins) may be activated during ketosis (Figure 2). Consequently, EKSs-evoked ketosis (increase in blood βHB levels) may influence all of above mentioned (e.g., mTOR-, AMPK- and SIRTs-evoked) downstream signaling pathways and modulatory effects, which can lead to generation of alleviating effects (e.g., anti-inflammatory effects) on age-related processes (aging hallmarks) (Figure 1 and Figure 2). Moreover, theoretically, EKSs-generated modulation of these signaling pathways and effects may be able to improve symptoms and/or delay development of not only aging-related hallmarks (such as changes in activity of nutrient sensing pathways, shortening of telomere, genomic instability, epigenetic alterations, mitochondrial dysfunction, altered intercellular communication, cellular senescence, loss of proteostasis and stem cell exhaustion), but also age-associated neurodegenerative diseases, and to extend lifespan (through both increased βHB level- and decreased glucose level-evoked changes in activity of several signaling pathways) (Figure 1 and Figure 2).

### 3.2. Beneficial Effects of EKSs-Evoked Ketosis (βHB) on Lifespan, Aging, Age-Related Diseases, as Well as Learning and Memory Dysfunctions

Administration of βHB generated anti-aging and life-extending effects in *C. elegans* [22,60]. This result suggests that lifespan extension by βHB may also be mediated in mammals through signaling pathways similar to *C. elegans* [60,294], likely by activation of AMPK/SIRT1/mTOR/FOXOs/Nrf2 pathways, HDAC inhibition (and related increase in FOXOs activity) or reduction of insulin signaling pathway activity (Figure 1 and Figure 2). Indeed, for example, it was demonstrated that inhibition of IIS pathways, thereby activation of FOXOs are important processes for lifespan extension [295] and FOXO3A gene is strongly associated with human longevity [296]. Increase in autophagy by caloric (or dietary) restriction can enhance lifespan not only in *C. elegans,* but also in mammals through similar pathways, which may also be activated by administration of EKSs, such as KEs and KSs. For example, in mammals, this effect may be mediated through βHB-evoked inhibition of mTOR activity, activation of FOXOs (via both activation of SIRT1 and direct inhibition of Akt), and ketone body metabolism-evoked decrease in blood glucose and insulin levels, which also decrease the activity of IIS pathways [52,77,180,297,298]. Moreover, long-lived animals showed decrease in mitochondrial ROS production [299] suggesting both inverse correlation between longevity and mitochondrial ROS production (and mitochondrial DNA damage) [52,299] and βHB-evoked enhancement of longevity (lifespan) (Figure 1 and Figure 2). It was also suggested that ketogenic diet (likely through ketogenic diet-generated ketosis/elevated blood βHB, at least partly) can reduce midlife mortality [300], extends longevity and healthspan in adult mice [51], increased lifespan in *Kcna1*-null mice [301] and decreased senescence may be partly through β-hydroxybutyrylation-evoked decrease in p53 activity (in addition, β-hydroxybutyrylation also can attenuate acetylation of p53, because β-hydroxybutyrylation interferes with acetylation) [302]. These results suggest that βHB-generated activation of different signaling pathways may have a role in modulation of aging processes, thereby both lifespan and healthspan. Indeed, it was demonstrated that βHB can alleviate cellular senescence through increased autophagy and decreased plasma insulin level and inflammatory processes in male rats [303], likely through AMPK/SIRT1 pathways (Figure 1). It has also been demonstrated that increased level of blood βHB can delay the age-related processes, for example, by inhibition of SASP, thereby senescence, likely through βHB/hnRNP A1-binding-evoked increase in binding of hnRNP A1 and Oct4 (embryonic stem cell regulator octamer-binding transcriptional factor 4) leading to stabilization of Oct4 mRNA (complex formation with Oct4 mRNA and upregulation of Oct4 expression) and SIRT1 mRNAs [59,304]. βHB-evoked activation of Oct4 not only triggers (maintains) quiescent state of cells (e.g., AMPK activation and mTOR inhibition), but also decreases induction of senescent state of cells (e.g., reduction of the blood level of a pro-senescence marker IL-1α and SASP expression) leading to protection of cells against senescence, and likely, induction of autophagy [59]. These results above suggest that, indeed, EKSs(βHB)-evoked ketosis can alleviate aging-processes (aging hallmarks), at least theoretically, through βHB-evoked activation of AMPK/SIRT1 or SIRT3 downstream signaling pathways (e.g., βHB/HCAR2/AMPK/SIRT1/NF-κB pathway), inhibition of mTOR- (e.g., βHB/HCAR2/AMPK/mTOR pathway) and NLRP3/IL-1R-generated effects, HDAC inhibition, β-hydroxybutyrylation and hnRNP A1-binding (Figure 1 and Figure 2) leading to improved healthspan, delayed aging, thereby extended lifespan.

A great deal of evidence suggests that progression of aging processes by age can lead to not only emergence of aging hallmarks, but also enhanced risk for development of neurodegenerative diseases and impaired learning and memory functions through, for example, mitochondrial dysfunction, epigenetic alterations and enhanced inflammation, which processes may be alleviated by EKSs-generated ketosis (βHB) (Figure 1 and Figure 2). For example, impaired mitochondrial functions, increased oxidative stress and neuronal injury were demonstrated in different CNS diseases, such as Alzheimer’s disease, Parkinson’s disease and amyotrophic lateral sclerosis [305,306,307,308]. Moreover, mitochondrial dysfunction-evoked increase in ROS level may enhance inflammatory processes [309,310], leading to impaired cognitive functions, for example in patients with neurodegenerative diseases (e.g., Alzheimer’s disease) [311,312,313]. It has been suggested that ketogenic diet- and EKSs-evoked ketosis can improve or prevent impaired cognitive functions, learning and memory, for example, via enhanced mitochondrial respiration and antioxidant mechanisms [49,314,315,316,317]. Indeed, not only ketogenic diet (and related ketosis) and βHB, but also KE, KS and MCT supplementation improved cognitive functions, learning and memory, as well as their age-related decline in animal models of Alzheimer’s disease and patients with Alzheimer’s disease or mild cognitive impairment [32,43,47,50,317,318,319,320] (Table 1), in a mouse model of Angelman syndrome [41] and in old animals and elderly humans [321,322]. EKSs may exert these beneficial effects via increased ketone body level, which can improve mitochondrial functions. For example, increased level of βHB can compensate glucose hypometabolism-generated decrease in energy source in human and restore ATP synthesis [16,289,318,319,323]. In fact, glucose hypometabolism may contribute to the development of, for example, Alzheimer’s disease [324,325]. It has also been demonstrated that MCT supplementation-evoked improvement in cognitive functions was observed in patients with mild to moderate Alzheimer’s disease or mild cognitive impairment without apolipoprotein E (*APOE*) ε4 allele [326,327], but the mechanism of action of *APOE*-*ε*4 status on MCT/ketosis-generated alleviating effects was not identified. Moreover, improved learning and memory was also demonstrated in relation to ketone bodies-evoked decrease in both oxidative stress and intracellular Aβ_42_ accumulation, and increased mitochondrial complex I activity in models of Alzheimer’s disease [50,328,329] (Table 1). It was demonstrated that βHB can protect neurons and alleviate symptoms in models of not only Alzheimer’s disease, but also Parkinson’s disease [328,330], likely via improvement of mitochondrial function (e.g., by increased ATP synthesis) and activation of other neuroprotective mechanisms, leading to improvement (or protection) in neurodegeneration, motor functions (e.g., tremor) and impaired cognition [258,259,328,331]. Moreover, indeed, βHB administration can decrease aggregation of α-synuclein and delay the toxicity of Aβ [60]. Ketogenic diet- and EKSs-generated ketosis, βHB or the Deanna protocol, containing (among others) MCTs, can also generate alleviating effects on (i) motor neurons and motor performance in preclinical rodent models, such as animal models of amyotrophic lateral sclerosis [48,332,333,334,335,336] and (ii) dopaminergic neurons and motor performance in animal models of Parkinson’s disease [55,258] likely through improved mitochondrial function and ATP synthesis (Table 1). Dysregulation of different neurotransmitter systems may have a role in the pathophysiology of neurodegenerative diseases, for example, in animal models and patients with impaired motor function (e.g., dopaminergic dysfunction; GABA and glutamate imbalance) [337,338,339,340], Parkinson’s disease (e.g., decrease in serotonin level and increase in glutamatergic transmission), Alzheimer’s disease (decreased cholinergic neurotransmission) and both Alzheimer’s disease and Parkinson’s disease (deficits in dopaminergic signaling) [337,339,341,342,343]. Moreover, dysfunctions in neurotransmitter systems (e.g., GABAergic, glutamatergic and cholinergic) can lead to impaired learning and memory [340,342,344]. It has also been demonstrated that dysregulation of acetylation and deacetylation can lead to neurodegenerative diseases (such as Alzheimer’s disease, Parkinson’s diseases, amyotrophic lateral sclerosis) and learning and memory deficits [345,346,347,348]. Moreover, HDAC inhibitors can improve symptoms or impede development of Parkinson’s disease, Alzheimer’s disease, amyotrophic lateral sclerosis and restore learning and memory functions [347,349,350,351,352]. Low BDNF levels were demonstrated in patients with Alzheimer’s disease, which decrease in BDNF level correlates with loss of cognitive functions [353,354], suggesting that ketosis (elevated blood βHB levels) can exert its beneficial effects on Alzheimer’s disease and cognitive functions, among others, through HDAC/BDNF system leading to enhancement of alleviating BDNF effects (e.g., by stimulation of hippocampal neurogenesis) [355]. Thus, EKSs (via ketosis/βHB) can exert alleviating effects on neurodegenerative diseases, learning and memory functions through modulation of not only mitochondrial functions and inflammatory processes, but also neurotransmitter systems and via epigenetic modification (Figure 2). Indeed, for example, it was suggested that EKSs may be able to prevent or improve neurodegenerative diseases and learning and memory, among others, through HDAC inhibition [30].

HCAR2 ligands can generate alleviating effects on Parkinson’s disease, Alzheimer’s disease, impaired learning, memory and motor functions, as well as amyotrophic lateral sclerosis via anti-inflammatory effects [43,50,57,258], suggesting that EKSs-evoked ketosis (βHB) exerts its alleviating effects on learning, memory, as well as age and age-related diseases through βHB/HCAR2-evoked downstream signaling (Figure 2). Indeed, previous studies show that ketosis (βHB) may evoke therapeutic effects in the treatment of Alzheimer’s disease, Parkinson’s disease and amyotrophic lateral sclerosis and enhance learning and memory through anti-inflammatory effects induced by HCAR2 [50,55,57,58,275,279]. It was also demonstrated that enhanced expression of proinflammatory cytokines and oxidative stress have a role in the development of Alzheimer’s disease [276,356,357], Parkinson’s disease [55,276,356,357], amyotrophic lateral sclerosis [356,357,358], impaired motor functions [337,359] and impairment of learning and memory [309,310,360]. Thus, ketosis may also improve symptoms of neurodegenerative diseases, motor, learning and memory dysfunctions through anti-inflammatory and anti-oxidative effects via HCAR2 [50,275,361] (Figure 2). It has been demonstrated that SIRT1 levels were decreased in neurodegenerative diseases, such as Alzheimer’s disease and Parkinson’s disease [97,362] suggesting alleviating effects of SIRT1 activation-modulated pathway(s) in the treatment of neurodegenerative diseases [363]. It was also suggested that activation of SIRT1-dependent pathways can modulate learning and memory by which ketone bodies may be able to improve both learning and memory functions [327]. Indeed, overexpression of SIRT1 was protective against learning and memory impairment in animal models of Alzheimer’s disease [364,365] and increased SIRT1 activity could promote memory processes, whereas SIRT1 knockout animals showed impaired cognitive abilities [366,367]. Moreover, activation of SIRT1 generated protective influences in mouse models of amyotrophic lateral sclerosis (e.g., enhanced biogenesis of mitochondria and suppressed deterioration of motor neurons) [94,368,369], preserved dopaminergic neurons in a mouse model of Parkinson’s disease [370] and evoked protection against Aβ plaque formation in mouse models of Alzheimer’s disease [94,371] likely via, for example, SIRT1/PGC1-α/MnSOD pathway [173,372]. In fact, it has been demonstrated that PGC1-α-deficiency may be in connection with neurodegenerative lesions [373], and decreased PGC1-α expression may be one of the most important factors in the development of both Parkinson’s disease [374,375] and Alzheimer’s disease [174,376]. Moreover, PPARγ agonist pioglitazone (an antidiabetic agent) and overexpression of PGC1-α were able to improve symptoms of amyotrophic lateral sclerosis in mouse models [377,378] and other PPARγ agonists can improve not only symptoms of neurodegenerative diseases (e.g., Parkinson’s disease, Alzheimer’s disease and amyotrophic lateral sclerosis), but also impaired cognitive functions, learning and memory [379,380]. As oxidative stress has a role in the pathophysiology of neurodegenerative diseases, such as Parkinson’s disease, Nrf2 thereby, for example, AMPK/SIRT1/Nrf2 pathway may be an important therapeutic target in the treatment of these diseases [381,382]. Moreover, it was also suggested that activation of SIRT3/PGC1-α/MnSOD pathways could also generate alleviating effect on Parkinson’s disease, Alzheimer’s disease, and amyotrophic lateral sclerosis [383,384,385]. Consequently, indeed, EKSs-generated ketosis (βHB) can alleviate or delay development of neurodegenerative diseases, and improve learning and memory dysfunctions likely through different βHB/HCAR2/AMPK-modulated downstream signaling pathways (Figure 2).

## 4. Conclusions

A great deal of evidence suggests that EKSs-generated ketosis may improve healthspan, therefore can delay ageing and the onset of age-related neurodegenerative diseases, as well as learning and memory dysfunctions through neuroprotective effects. In spite of the overwhelming amount of promising mechanistic findings, only a limited number of studies focused on and demonstrated the beneficial effects of EKSs-evoked ketosis on lifespan, aging-processes, age-related diseases and impaired learning and memory functions. However, their beneficial effects on healthspan and lifespan—likely through improving mitochondrial functions, anti-oxidant effects, anti-inflammatory influences, and modulation of histone and non-histone acetylation, as well as neurotransmitter systems-, can be hypothesized. Indeed, it has been suggested that EKSs-evoked ketosis may alter the activity of different downstream signaling pathways (e.g., AMPK-, SIRTs- and mTOR-modulated pathways) and modulatory effects, through which not only senotherapeutic drugs, but also ketosis (βHB) can improve symptoms and delay development of age-related hallmarks, age-associated neurodegenerative diseases and learning and memory dysfunctions, and extend lifespan. Consequently, administration of EKSs may be a potential therapeutic tool as an adjuvant therapeutics in combination with different therapeutic drugs (such as metformin and rapamycin) for regenerative medicine to enhance effectivity of drugs to rejuvenate aging hallmarks, decrease the risk for age-related neurodegenerative diseases and increase the healthspan of the aging human population. However, modulating ageing processes and related diseases by administration of EKSs needs careful attention, because insufficient clinical data is available currently on its positive effects, efficacy and safety, regarding this specific application. Thus, long-term studies are needed to investigate the exact mechanisms of action by which EKSs-evoked ketosis modulate aging processes, age-related diseases, learning and memory functions, healthspan and lifespan. Moreover, in order to develop effective treatments for patients with different age-related diseases more studies are needed to identify the most effective doses, administration routes, treatment duration and different formulations of EKSs.

## Figures and Tables

**Table 1:** Beneficial effects of beta-hydroxybutyrate (βHB), ketone esters (KEs) and medium chain triglycerides (MCTs) on neurodegenerative diseases as well as impaired motor, memory and learning functions in in vivo studies.

Name (Components)	Dose and Route of Administration	Treatment Duration	Model Organism (Species)	Significant Increase in Blood βHB Level	Main Findings	Ref.
Beta-hydroxybutyrate (βHB)						
βHB (DL-β-Hydroxybutyric acid sodium salt)	1.5 mmol/kg/day (subcutaneous administration, 0.25 μL/h)	4 weeks	A mouse model of Alzheimer’s disease (5XFAD)	No data	Improved learning and memory; attenuated Aβ accumulation	[50]
βHB + acetoacetate	600 mg βHB/kg/day + 150 mg acetoacetate/kg/day (subcutaneous injection)	2 months	A mouse model of Alzheimer’s disease (APPSwInd)	Yes	Improved cognitive performance; reduced Aβ accumulation	[329]
βHB	0.4, 0.8, or 1.6 mmol/kg/day (subcutaneous administration, 1 μL/h)	28 days	LPS-induced Parkinson’s disease rat model	No data	Beneficial effects on motor dysfunction; protection of dopaminergic neurons	[55]
βHB (D-βHB)	0.4, 0.8, or 1.6 mmol/kg/day (subcutaneous administration, 1 μL/h)	1 week	MPTP-induced Parkinson’s disease mouse model	Yes	Improved motor performance; decrease in MPTP-induced dopaminergic neurodegeneration	[258]
Ketone esters (KEs)						
KE (*R*,*S*-1,3-butanediol acetoacetate diester: BD-AcAc_2_; standard rodent chow mixed at 10% BD-AcAc_2_ by volume and 1% saccharin)	*Ad libitum* (oral intake)	8 weeks	A mouse model of Angelman syndrome (*UBE3A^tm1Alb/J^* null mutation mice)	Yes	Improved motor coordination, learning and memory	[41]
KE (comprised of D-β-hydroxybutyrate and (*R*)-1,3-butanediol; 125 g KE/1000 g diet)	Animals were fed a 4 to 5 g pellet/animal at approximately 06:00 hours each day (oral intake)	8 months	A mouse model of Alzheimer’s disease (3xTgAD)	Yes	Improvements in performance on learning and memory tests; decreased Aβ and hyperphosphorylated tau deposition	[43]
KE [ketone monoester, (*R*)-3-hydroxybutyl (*R*)-3-hydroxybutyrate] + MCT and coconut oil (CO) mixture (4:3)	Normal diet + 28.7 g of the KE thrice daily + 165 mL/day of the MCT/CO mixture (oral intake)	20 months	A patient with Alzheimer’s disease dementia	Yes	Improving behavior as well as cognitive and daily-activity performance	[47]
Medium chain triglycerides (MCTs)						
MCT (97% caprylic acid + 3% capric acid; a normal diet supplemented with 5.5% MCT)	Dogs were fed once/day for about one hour; about 200 g supplemented diet/day/animal (oral intake)	8 months	Aged dogs	Yes	Improvements in learning ability and attention	[322]
MCT (the diet was mixed with Deanna protocol/DP at 22% by weight; DP contained 10% MCT high in caprylic acid)	*Ad libitum* (oral intake)	6–10 weeks	A mouse model of Amyotrophic lateral sclerosis (SOD1-G93A)	No	Better motor performance, improved (lower) neurological scores and extended survival time	[332]
MCT (a diet in which 35% of the calories was derived from triheptanoin)	*Ad libitum* (oral intake)	24 weeks	A mouse model of Amyotrophic lateral sclerosis (SOD1-G93A)	Yes	Protection against motor neuron loss; improved motor function	[48]
MCT [a diet containing 10% (*w*/*w*) caprylic acid]	*Ad libitum*; about 3 g diet/day was consumed/animal (oral intake)	About 12 weeks	A mouse model of Amyotrophic lateral sclerosis (SOD1-G93A)	Yes	Protection against motor neuron loss; improved motor function	[336]
MCT (NeoBee 895, >95% of the fatty acids are caprylic acid; the remainder consists of caproic and capric acids)	40 mL MCT (oral intake)	Single administration	Adult subjects with Alzheimer’s disease or mild cognitive impairment	Yes	Improvement in cognitive functions (in patients without *APOE* ε4 allele)	[327]
MCT (AC-1202, an MCT composed of glycerin and, almost entirely, caprylic acid, NeoBee 895)	Normal diet + 20 g MCT/day/patient (oral intake)	3 months	Humans with mild to moderate Alzheimer’s disease	Yes	Improvement in cognitive performance (in patients without *APOE* ε4 allele)	[326]
MCT (50 g Ketogenic meal, Ketonformula containing 20 g of MCTs: 15 g caprylic acid + 5 g capric acid)	50 g ketogenic meal (oral intake)	Single administration	Humans; elderly, non-demented	Yes	Positive effects on working memory, visual attention, and task switching	[321]
MCT (MCT drink: a 12% emulsion of Captex 355, containing 60% caprylic acid and 40% capric acid)	Normal diet + 15 g MCT twice/day/patient in a ketogenic drink (oral intake)	6 months	Humans; aged participants with mild cognitive impairment	Yes	Improved executive function, memory, and language	[32]
MCT (MCT oil, Nestle™)	Normal diet + 56 g MCT/day/patient (oral intake)	24 weeks	Humans; adults with mild cognitive impairment	Yes	Improved memory	[320]

Abbreviations: 5XFAD, β-amyloid precursor protein and presenilin-1 double-transgenic mouse; Aβ, amyloid β; *APOE*, apolipoprotein E; APPSwInd, a transgenic mouse, express a mutant form of the human amyloid protein precursor (APP) with the APP KM670/671NL (Swedish) and APP V717F (Indiana) mutations; LPS, lipopolysaccharide; MPTP, 1-methyl-4-phenyl-1,2,3,6-tetrahydropyridine; *UBE3A^tm1Alb^*, a mouse with *Ube3A* (ubiquitin protein ligase E3A) knock-out mutation; 3xTgAD, a mouse with APP KM670/671NL (Swedish), MAPT P301L and PSEN1 M146V mutations; SOD1-G93A, a transgenic mouse with a G93A mutant form of human superoxide dismutase (SOD1).

## Data Availability

Not applicable.

## Abbreviations

Aβ: amyloid-β; ACCs, acetyl-CoA carboxylases; Akt, Akt kinase/protein kinase B; AMPK, AMP activated serine-threonine protein kinase; BDNF, brain-derived neurotrophic factor; βHB, beta-hydroxybutyrate; CNS, central nervous system; EKSs, exogenous ketogenic supplements; ER, endoplasmic reticulum; ETC, electron transport chain; FOXOs, Forkhead box Os; HATs, histone acetyltransferases; HCAR2, hydroxycarboxylic acid receptor 2; HDACs, histone deacetylases; hnRNP A1, heterogeneous nuclear ribonucleoprotein A1; IGF 1, insulin-like growth factor 1; IIS pathway, insulin/insulin-like growth factor (IGF) 1 pathway; IL-1β, interleukin-1β; IL-1R, IL-1 receptor; KE, ketone ester; KS, ketone salt; MCT, medium chain triglyceride; miRNAs, microRNAs; MnSOD, manganese superoxide dismutase; mPT pore, mitochondrial permeability transition pore; mTOR, mechanistic target of rapamycin; NAD^+^, nicotinamide adenine dinucleotide; NF-κB, nuclear factor kappa-light-chain-enhancer of activated B cells; NLRP3, NOD-like receptor pyrin domain 3; Nrf2, nuclear factor erythroid 2-related factor 2; Oct4, embryonic stem cell regulator octamer-binding transcriptional factor 4; p53, transcription factor tumor suppressor protein 53; PARP-1, poly(ADP-ribose)-polymerase-1; PGC-1α, peroxisome proliferator-activated receptor gamma (PPARγ) coactivator-1α; ROS, reactive oxygen species; SASP, senescence associated secretory phenotype; SIRT, Sirtuin; SOD, superoxide dismutase; SREBP1, sterol regulatory element-binding protein 1; TNF-α, tumor necrosis factor-α; UCP, uncoupling protein; ULK1, Uncoordinated/Unc-51-like kinase 1.

## References

[1] Campisi J., Kapahi P., Lithgow G.J., Melov S., Newman J.C., Verdin E. (2019). From discoveries in ageing research to therapeutics for healthy ageing. Nature.

[2] Li Z., Zhang Z., Ren Y., Wang Y., Fang J., Yue H., Ma S., Guan F. (2021). Aging and age-related diseases: From mechanisms to therapeutic strategies. Biogerontology.

[3] Sen A., Capelli V., Husain M. (2018). Cognition and dementia in older patients with epilepsy. Brain.

[4] United Nations, Department of Economic and Social Affairs, Population Division (2019). World Population Ageing 2019: Highlights.

[5] Drygalski K., Fereniec E., Koryciński K., Chomentowski A., Kiełczewska A., Odrzygóźdź C., Modzelewska B. (2018). Resveratrol and Alzheimer’s disease. From molecular pathophysiology to clinical trials. Exp. Gerontol..

[6] Yang C., Zhang W., Dong X., Fu C., Yuan J., Xu M., Liang Z., Qiu C., Xu C. (2020). A natural product solution to aging and aging-associated diseases. Pharmacol. Ther..

[7] De Magalhães J.P., Stevens M., Thornton D. (2017). The Business of Anti-Aging Science. Trends Biotechnol..

[8] Anisimov V.N., Zabezhinski M.A., Popovich I.G., Piskunova T.S., Semenchenko A.V., Tyndyk M.L., Yurova M.N., Rosenfeld S.V., Blagosklonny M.V. (2011). Rapamycin increases lifespan and inhibits spontaneous tumorigenesis in inbred female mice. Cell Cycle.

[9] Bitto A., Ito T.K., Pineda V.V., LeTexier N.J., Huang H.Z., Sutlief E., Tung H., Vizzini N., Chen B., Smith K. (2016). Transient rapamycin treatment can increase lifespan and healthspan in middle-aged mice. Elife.

[10] Mannick J.B., Morris M., Hockey H.P., Roma G., Beibel M., Kulmatycki K., Watkins M., Shavlakadze T., Zhou W., Quinn D. (2018). TORC1 inhibition enhances immune function and reduces infections in the elderly. Sci. Transl. Med..

[11] Bannister C.A., Holden S.E., Jenkins-Jones S., Morgan C.L., Halcox J.P., Schernthaner G., Mukherjee J., Currie C.J. (2014). Can people with type 2 diabetes live longer than those without? A comparison of mortality in people initiated with metformin or sulphonylurea monotherapy and matched, non-diabetic controls. Diabetes Obes. Metab..

[12] Barzilai N., Crandall J.P., Kritchevsky S.B., Espeland M.A. (2016). Metformin as a Tool to Target Aging. Cell Metab..

[13] Bonkowski M.S., Sinclair D.A. (2016). Slowing ageing by design: The rise of NAD^+^ and sirtuin-activating compounds. Nat. Rev. Mol. Cell Biol..

[14] Kane A.E., Sinclair D.A. (2018). Sirtuins and NAD^+^ in the Development and Treatment of Metabolic and Cardiovascular Diseases. Circ. Res..

[15] Kirkland J.L., Tchkonia T., Zhu Y., Niedernhofer L.J., Robbins P.D. (2017). The Clinical Potential of Senolytic Drugs. J. Am. Geriatr. Soc..

[16] Brownlow M.L., Jung S.H., Moore R.J., Bechmann N., Jankord R. (2017). Nutritional Ketosis Affects Metabolism and Behavior in Sprague-Dawley Rats in Both Control and Chronic Stress Environments. Front. Mol. Neurosci..

[17] Brunengraber H. (1997). Potential of ketone body esters for parenteral and oral nutrition. Nutrition.

[18] Clarke K., Tchabanenko K., Pawlosky R., Carter E., Knight N.S., Murray A.J., Cochlin L.E., King M.T., Wong A.W., Roberts A. (2012). Oral 28-day and developmental toxicity studies of (*R*)-3-hydroxybutyl (*R*)-3-hydroxybutyrate. Regul. Toxicol. Pharmacol..

[19] Clarke K., Tchabanenko K., Pawlosky R., Carter E., Todd King M., Musa-Veloso K., Ho M., Roberts A., Robertson J., Vanitallie T.B. (2012). Kinetics, safety and tolerability of (*R*)-3-hydroxybutyl (*R*)-3-hydroxybutyrate in healthy adult subjects. Regul. Toxicol. Pharmacol..

[20] Schönfeld P., Wojtczak L. (2016). Short- and medium-chain fatty acids in energy metabolism: The cellular perspective. J. Lipid Res..

[21] Kovács Z., Brunner B., D’Agostino D.P., Ari C. (2021). Age- and Sex-Dependent Modulation of Exogenous Ketone Supplement-Evoked Effects on Blood Glucose and Ketone Body Levels in Wistar Albino Glaxo Rijswijk Rats. Front. Neurosci..

[22] Achanta L.B., Rae C.D. (2017). β-Hydroxybutyrate in the Brain: One Molecule, Multiple Mechanisms. Neurochem. Res..

[23] Koppel S.J., Swerdlow R.H. (2018). Neuroketotherapeutics: A modern review of a century-old therapy. Neurochem. Int..

[24] Newman J.C., Verdin E. (2014). Ketone bodies as signaling metabolites. Trends Endocrinol. Metab..

[25] Soto-Mota A., Norwitz N.G., Clarke K. (2020). Why a d-β-hydroxybutyrate monoester?. Biochem. Soc. Trans..

[26] Ari C., Kovács Z., Juhasz G., Murdun C., Goldhagen C.R., Koutnik A.P., Poff A.M., Kesl S.L., D’Agostino D.P. (2016). Exogenous Ketone Supplements Reduce Anxiety-Related Behavior in Sprague-Dawley and Wistar Albino Glaxo/Rijswijk Rats. Front. Mol. Neurosci..

[27] Kesl S.L., Poff A.M., Ward N.P., Fiorelli T.N., Ari C., Van Putten A.J., Sherwood J.W., Arnold P., D’Agostino D.P. (2016). Effects of exogenous ketone supplementation on blood ketone, glucose, triglyceride, and lipoprotein levels in Sprague-Dawley rats. Nutr. Metab..

[28] Myette-Côté É., Neudorf H., Rafiei H., Clarke K., Little J.P. (2018). Prior ingestion of exogenous ketone monoester attenuates the glycaemic response to an oral glucose tolerance test in healthy young individuals. J. Physiol..

[29] Stubbs B.J., Cox P.J., Evans R.D., Santer P., Miller J.J., Faull O.K., Magor-Elliott S., Hiyama S., Stirling M., Clarke K. (2017). On the Metabolism of Exogenous Ketones in Humans. Front. Physiol..

[30] Hashim S.A., VanItallie T.B. (2014). Ketone body therapy: From the ketogenic diet to the oral administration of ketone ester. J. Lipid Res..

[31] McDonald T.J., Cervenka M.C. (2019). Lessons learned from recent clinical trials of ketogenic diet therapies in adults. Curr. Opin. Clin. Nutr. Metab. Care.

[32] Fortier M., Castellano C.A., St-Pierre V., Myette-Côté É., Langlois F., Roy M., Morin M.C., Bocti C., Fulop T., Godin J.P. (2021). A ketogenic drink improves cognition in mild cognitive impairment: Results of a 6-month RCT. Alzheimers Dement..

[33] Soto-Mota A., Norwitz N.G., Evans R., Clarke K., Barber T.M. (2021). Exogenous ketosis in patients with type 2 diabetes: Safety, tolerability and effect on glycaemic control. Endocrinol. Diabetes Metab..

[34] Camberos-Luna L., Massieu L. (2020). Therapeutic strategies for ketosis induction and their potential efficacy for the treatment of acute brain injury and neurodegenerative diseases. Neurochem. Int..

[35] Han Y.M., Ramprasath T., Zou M.H. (2020). β-hydroxybutyrate and its metabolic effects on age-associated pathology. Exp. Mol. Med..

[36] Kovács Z., D’Agostino D.P., Dobolyi A., Ari C. (2017). Adenosine A1 Receptor Antagonism Abolished the Anti-seizure Effects of Exogenous Ketone Supplementation in Wistar Albino Glaxo Rijswijk Rats. Front. Mol. Neurosci..

[37] Branco A.F., Ferreira A., Simões R.F., Magalhães-Novais S., Zehowski C., Cope E., Silva A.M., Pereira D., Sardão V.A., Cunha-Oliveira T. (2016). Ketogenic diets: From cancer to mitochondrial diseases and beyond. Eur. J. Clin. Investig..

[38] Kim D.Y., Simeone K.A., Simeone T.A., Pandya J.D., Wilke J.C., Ahn Y., Geddes J.W., Sullivan P.G., Rho J.M. (2015). Ketone bodies mediate antiseizure effects through mitochondrial permeability transition. Ann. Neurol..

[39] Kovács Z., D’Agostino D.P., Diamond D., Kindy M.S., Rogers C., Ari C. (2019). Therapeutic Potential of Exogenous Ketone Supplement Induced Ketosis in the Treatment of Psychiatric Disorders: Review of Current Literature. Front. Psychiatry.

[40] Berk B.A., Law T.H., Packer R.M.A., Wessmann A., Bathen-Nöthen A., Jokinen T.S., Knebel A., Tipold A., Pelligand L., Meads Z. (2020). A multicenter randomized controlled trial of medium-chain triglyceride dietary supplementation on epilepsy in dogs. J. Vet. Intern. Med..

[41] Ciarlone S.L., Grieco J.C., D’Agostino D.P., Weeber E.J. (2016). Ketone ester supplementation attenuates seizure activity, and improves behavior and hippocampal synaptic plasticity in an Angelman syndrome mouse model. Neurobiol. Dis..

[42] D’Agostino D.P., Pilla R., Held H.E., Landon C.S., Puchowicz M., Brunengraber H., Ari C., Arnold P., Dean J.B. (2013). Therapeutic ketosis with ketone ester delays central nervous system oxygen toxicity seizures in rats. Am. J. Physiol. Regul. Integr. Comp. Physiol..

[43] Kashiwaya Y., Bergman C., Lee J.H., Wan R., King M.T., Mughal M.R., Okun E., Clarke K., Mattson M.P., Veech R.L. (2013). A ketone ester diet exhibits anxiolytic and cognition-sparing properties, and lessens amyloid and tau pathologies in a mouse model of Alzheimer’s disease. Neurobiol. Aging.

[44] Kovács Z., D’Agostino D.P., Ari C. (2018). Anxiolytic Effect of Exogenous Ketone Supplementation Is Abolished by Adenosine A1 Receptor Inhibition in Wistar Albino Glaxo/Rijswijk Rats. Front. Behav. Neurosci..

[45] Ari C., Zippert M., D’Agostino D.P. (2018). Neuroregeneration improved by ketones. FASEB J..

[46] Ari C., Pilla R., D’Agostino D., Ross Watson R., Preedy V.R. (2015). Nutritional/metabolic therapies in animal models of amyotrophic lateral sclerosis, Alzheimer’s disease, and seizures. Bioactive Nutraceuticals and Dietary Supplements in Neurological and Brain Disease.

[47] Newport M.T., VanItallie T.B., Kashiwaya Y., King M.T., Veech R.L. (2015). A new way to produce hyperketonemia: Use of ketone ester in a case of Alzheimer’s disease. Alzheimers Dement..

[48] Tefera T.W., Wong Y., Barkl-Luke M.E., Ngo S.T., Thomas N.K., McDonald T.S., Borges K. (2016). Triheptanoin Protects Motor Neurons and Delays the Onset of Motor Symptoms in a Mouse Model of Amyotrophic Lateral Sclerosis. PLoS ONE.

[49] Maalouf M., Rho J.M., Mattson M.P. (2009). The neuroprotective properties of calorie restriction, the ketogenic diet, and ketone bodies. Brain Res. Rev..

[50] Wu Y., Gong Y., Luan Y., Li Y., Liu J., Yue Z., Yuan B., Sun J., Xie C., Li L. (2020). BHBA treatment improves cognitive function by targeting pleiotropic mechanisms in transgenic mouse model of Alzheimer’s disease. FASEB J..

[51] Roberts M.N., Wallace M.A., Tomilov A.A., Zhou Z., Marcotte G.R., Tran D., Perez G., Gutierrez-Casado E., Koike S., Knotts T.A. (2017). A Ketogenic Diet Extends Longevity and Healthspan in Adult Mice. Cell Metab..

[52] Veech R.L., Bradshaw P.C., Clarke K., Curtis W., Pawlosky R., King M.T. (2017). Ketone bodies mimic the life span extending properties of caloric restriction. IUBMB Life.

[53] Newman J.C., Verdin E. (2014). β-hydroxybutyrate: Much more than a metabolite. Diabetes Res. Clin. Pract..

[54] Rahman M., Muhammad S., Khan M.A., Chen H., Ridder D.A., Müller-Fielitz H., Pokorná B., Vollbrandt T., Stölting I., Nadrowitz R. (2014). The β-hydroxybutyrate receptor HCA2 activates a neuroprotective subset of macrophages. Nat. Commun..

[55] Fu S.P., Wang J.F., Xue W.J., Liu H.M., Liu B.R., Zeng Y.L., Li S.N., Huang B.X., Lv Q.K., Wang W. (2015). Anti-inflammatory effects of BHBA in both in vivo and in vitro Parkinson’s disease models are mediated by GPR109A-dependent mechanisms. J. Neuroinflamm..

[56] Rezq S., Abdel-Rahman A.A. (2016). Central GPR109A Activation Mediates Glutamate-Dependent Pressor Response in Conscious Rats. J. Pharmacol. Exp. Ther..

[57] Graff E.C., Fang H., Wanders D., Judd R.L. (2016). Anti-inflammatory effects of the hydroxycarboxylic acid receptor 2. Metabolism.

[58] Wakade C., Chong R., Bradley E., Thomas B., Morgan J. (2014). Upregulation of GPR109A in Parkinson’s disease. PLoS ONE.

[59] Han Y.M., Bedarida T., Ding Y., Somba B.K., Lu Q., Wang Q., Song P., Zou M.H. (2018). β-Hydroxybutyrate Prevents Vascular Senescence through hnRNP A1-Mediated Upregulation of Oct4. Mol. Cell.

[60] Edwards C., Canfield J., Copes N., Rehan M., Lipps D., Bradshaw P.C. (2014). D-beta-hydroxybutyrate extends lifespan in *C. elegans*. Aging.

[61] Trevisan K., Cristina-Pereira R., Silva-Amaral D., Aversi-Ferreira T.A. (2019). Theories of Aging and the Prevalence of Alzheimer’s Disease. Biomed. Res. Int..

[62] Ascherio A., Schwarzschild M.A. (2016). The epidemiology of Parkinson’s disease: Risk factors and prevention. Lancet Neurol..

[63] Hou Y., Dan X., Babbar M., Wei Y., Hasselbalch S.G., Croteau D.L., Bohr V.A. (2019). Ageing as a risk factor for neurodegenerative disease. Nat. Rev. Neurol..

[64] Pandya V.A., Patani R. (2020). Decoding the relationship between ageing and amyotrophic lateral sclerosis: A cellular perspective. Brain.

[65] Broughton S., Partridge L. (2009). Insulin/IGF-like signalling, the central nervous system and aging. Biochem. J..

[66] Klement R.J., Fink M.K. (2016). Dietary and pharmacological modification of the insulin/IGF-1 system: Exploiting the full repertoire against cancer. Oncogenesis.

[67] Martin B., Mattson M.P., Maudsley S. (2006). Caloric restriction and intermittent fasting: Two potential diets for successful brain aging. Ageing Res. Rev..

[68] Santos J., Leitão-Correia F., Sousa M.J., Leão C. (2016). Dietary Restriction and Nutrient Balance in Aging. Oxid. Med. Cell Longev..

[69] López-Otín C., Blasco M.A., Partridge L., Serrano M., Kroemer G. (2013). The hallmarks of aging. Cell.

[70] Nugent S., Tremblay S., Chen K.W., Ayutyanont N., Roontiva A., Castellano C.A., Fortier M., Roy M., Courchesne-Loyer A., Bocti C. (2014). Brain glucose and acetoacetate metabolism: A comparison of young and older adults. Neurobiol. Aging.

[71] Schultz M.B., Sinclair D.A. (2016). When stem cells grow old: Phenotypes and mechanisms of stem cell aging. Development.

[72] Mathew R., Pal Bhadra M., Bhadra U. (2017). Insulin/insulin-like growth factor-1 signalling (IIS) based regulation of lifespan across species. Biogerontology.

[73] Cohen E., Bieschke J., Perciavalle R.M., Kelly J.W., Dillin A. (2006). Opposing activities protect against age-onset proteotoxicity. Science.

[74] Li J., Kim S.G., Blenis J. (2014). Rapamycin: One drug, many effects. Cell Metab..

[75] Saxton R.A., Sabatini D.M. (2017). mTOR Signaling in Growth, Metabolism, and Disease. Cell.

[76] Johnson S.C., Rabinovitch P.S., Kaeberlein M. (2013). mTOR is a key modulator of ageing and age-related disease. Nature.

[77] Menzies F.M., Fleming A., Caricasole A., Bento C.F., Andrews S.P., Ashkenazi A., Füllgrabe J., Jackson A., Jimenez Sanchez M., Karabiyik C. (2017). Autophagy and Neurodegeneration: Pathogenic Mechanisms and Therapeutic Opportunities. Neuron.

[78] Sen P., Shah P.P., Nativio R., Berger S.L. (2016). Epigenetic Mechanisms of Longevity and Aging. Cell.

[79] Carling D. (2004). The AMP-activated protein kinase cascade—A unifying system for energy control. Trends Biochem. Sci..

[80] Steinberg G.R., Kemp B.E. (2009). AMPK in Health and Disease. Physiol. Rev..

[81] Chung M.M., Nicol C.J., Cheng Y.C., Lin K.H., Chen Y.L., Pei D., Lin C.H., Shih Y.N., Yen C.H., Chen S.J. (2017). Metformin activation of AMPK suppresses AGE-induced inflammatory response in hNSCs. Exp. Cell Res..

[82] Salminen A., Hyttinen J.M., Kaarniranta K. (2011). AMP-activated protein kinase inhibits NF-κB signaling and inflammation: Impact on healthspan and lifespan. J. Mol. Med..

[83] Cantó C., Gerhart-Hines Z., Feige J.N., Lagouge M., Noriega L., Milne J.C., Elliott P.J., Puigserver P., Auwerx J. (2009). AMPK regulates energy expenditure by modulating NAD+ metabolism and SIRT1 activity. Nature.

[84] Kapahi P., Kaeberlein M., Hansen M. (2017). Dietary restriction and lifespan: Lessons from invertebrate models. Ageing Res. Rev..

[85] He W., Newman J.C., Wang M.Z., Ho L., Verdin E. (2012). Mitochondrial sirtuins: Regulators of protein acylation and metabolism. Trends Endocrinol. Metab..

[86] Gillum M.P., Kotas M.E., Erion D.M., Kursawe R., Chatterjee P., Nead K.T., Muise E.S., Hsiao J.J., Frederick D.W., Yonemitsu S. (2011). SirT1 regulates adipose tissue inflammation. Diabetes.

[87] Rodgers J.T., Lerin C., Haas W., Gygi S.P., Spiegelman B.M., Puigserver P. (2005). Nutrient control of glucose homeostasis through a complex of PGC-1alpha and SIRT1. Nature.

[88] Imai S., Guarente L. (2014). NAD+ and sirtuins in aging and disease. Trends Cell Biol..

[89] Morigi M., Perico L., Benigni A. (2018). Sirtuins in Renal Health and Disease. J. Am. Soc. Nephrol..

[90] Wątroba M., Dudek I., Skoda M., Stangret A., Rzodkiewicz P., Szukiewicz D. (2017). Sirtuins, epigenetics and longevity. Ageing Res. Rev..

[91] Cho S.H., Chen J.A., Sayed F., Ward M.E., Gao F., Nguyen T.A., Krabbe G., Sohn P.D., Lo I., Minami S. (2015). SIRT1 deficiency in microglia contributes to cognitive decline in aging and neurodegeneration via epigenetic regulation of IL-1β. J. Neurosci..

[92] Patel N.V., Gordon M.N., Connor K.E., Good R.A., Engelman R.W., Mason J., Morgan D.G., Morgan T.E., Finch C.E. (2005). Caloric restriction attenuates Abeta-deposition in Alzheimer transgenic models. Neurobiol. Aging.

[93] Wang J., Fivecoat H., Ho L., Pan Y., Ling E., Pasinetti G.M. (2010). The role of Sirt1: At the crossroad between promotion of longevity and protection against Alzheimer’s disease neuropathology. Biochim. Biophys. Acta.

[94] Kim D., Nguyen M.D., Dobbin M.M., Fischer A., Sananbenesi F., Rodgers J.T., Delalle I., Baur J.A., Sui G., Armour S.M. (2007). SIRT1 deacetylase protects against neurodegeneration in models for Alzheimer’s disease and amyotrophic lateral sclerosis. EMBO J..

[95] Marambaud P., Zhao H., Davies P. (2005). Resveratrol promotes clearance of Alzheimer’s disease amyloid-beta peptides. J. Biol. Chem..

[96] Luchsinger J.A., Tang M.X., Shea S., Mayeux R. (2002). Caloric intake and the risk of Alzheimer disease. Arch. Neurol..

[97] Julien C., Tremblay C., Emond V., Lebbadi M., Salem N., Bennett D.A., Calon F. (2009). Sirtuin 1 reduction parallels the accumulation of tau in Alzheimer disease. J. Neuropathol. Exp. Neurol..

[98] Oosterhof N., Dekens D.W., Lawerman T.F., van Dijk M. (2012). Yet another role for SIRT1: Reduction of α-synuclein aggregation in stressed neurons. J. Neurosci..

[99] Araki T., Sasaki Y., Milbrandt J. (2004). Increased nuclear NAD biosynthesis and SIRT1 activation prevent axonal degeneration. Science.

[100] Virgili M., Contestabile A. (2000). Partial neuroprotection of in vivo excitotoxic brain damage by chronic administration of the red wine antioxidant agent, trans-resveratrol in rats. Neurosci. Lett..

[101] Brunet A., Sweeney L.B., Sturgill J.F., Chua K.F., Greer P.L., Lin Y., Tran H., Ross S.E., Mostoslavsky R., Cohen H.Y. (2004). Stress-dependent regulation of FOXO transcription factors by the SIRT1 deacetylase. Science.

[102] Zhao L., Cao J., Hu K., He X., Yun D., Tong T., Han L. (2020). Sirtuins and their Biological Relevance in Aging and Age-Related Diseases. Aging Dis..

[103] Vaziri H., Dessain S.K., Ng Eaton E., Imai S.I., Frye R.A., Pandita T.K., Guarente L., Weinberg R.A. (2001). hSIR2(SIRT1) functions as an NAD-dependent p53 deacetylase. Cell.

[104] Prozorovski T., Schulze-Topphoff U., Glumm R., Baumgart J., Schröter F., Ninnemann O., Siegert E., Bendix I., Brüstle O., Nitsch R. (2008). Sirt1 contributes critically to the redox-dependent fate of neural progenitors. Nat. Cell Biol..

[105] Chang H.C., Guarente L. (2013). SIRT1 mediates central circadian control in the SCN by a mechanism that decays with aging. Cell.

[106] Yaku K., Okabe K., Nakagawa T. (2018). NAD metabolism: Implications in aging and longevity. Ageing Res. Rev..

[107] Blüher M., Kahn B.B., Kahn C.R. (2003). Extended longevity in mice lacking the insulin receptor in adipose tissue. Science.

[108] Kenyon C.J. (2010). The genetics of ageing. Nature.

[109] Greer E.L., Brunet A. (2009). Different dietary restriction regimens extend lifespan by both independent and overlapping genetic pathways in *C. elegans*. Aging Cell.

[110] Zhu Y., Liu X., Ding X., Wang F., Geng X. (2019). Telomere and its role in the aging pathways: Telomere shortening, cell senescence and mitochondria dysfunction. Biogerontology.

[111] Herrmann M., Pusceddu I., März W., Herrmann W. (2018). Telomere biology and age-related diseases. Clin. Chem. Lab. Med..

[112] Sahin E., DePinho R.A. (2012). Axis of ageing: Telomeres, p53 and mitochondria. Nat. Rev. Mol. Cell Biol..

[113] Bernardes de Jesus B., Vera E., Schneeberger K., Tejera A.M., Ayuso E., Bosch F., Blasco M.A. (2012). Telomerase gene therapy in adult and old mice delays aging and increases longevity without increasing cancer. EMBO Mol. Med..

[114] Shay J.W. (2016). Role of Telomeres and Telomerase in Aging and Cancer. Cancer Discov..

[115] Palacios J.A., Herranz D., De Bonis M.L., Velasco S., Serrano M., Blasco M.A. (2010). SIRT1 contributes to telomere maintenance and augments global homologous recombination. J. Cell Biol..

[116] Tiwari V., Wilson D.M. (2019). DNA Damage and Associated DNA Repair Defects in Disease and Premature Aging. Am. J. Hum. Genet..

[117] Foo M.X.R., Ong P.F., Dreesen O. (2019). Premature aging syndromes: From patients to mechanism. J. Dermatol. Sci..

[118] Hoeijmakers J.H. (2009). DNA damage, aging, and cancer. N. Engl. J. Med..

[119] Kauppila T.E.S., Bratic A., Jensen M.B., Baggio F., Partridge L., Jasper H., Grönke S., Larsson N.G. (2018). Mutations of mitochondrial DNA are not major contributors to aging of fruit flies. Proc. Natl. Acad. Sci. USA.

[120] Thanan R., Oikawa S., Hiraku Y., Ohnishi S., Ma N., Pinlaor S., Yongvanit P., Kawanishi S., Murata M. (2014). Oxidative stress and its significant roles in neurodegenerative diseases and cancer. Int. J. Mol. Sci..

[121] Feinberg A.P., Tycko B. (2004). The history of cancer epigenetics. Nat. Rev. Cancer.

[122] Kane A.E., Sinclair D.A. (2019). Epigenetic changes during aging and their reprogramming potential. Crit. Rev. Biochem. Mol. Biol..

[123] Burzynski S.R. (2005). Aging: Gene silencing or gene activation?. Med. Hypotheses.

[124] Ben-Avraham D., Muzumdar R.H., Atzmon G. (2012). Epigenetic genome-wide association methylation in aging and longevity. Epigenomics.

[125] Benayoun B.A., Pollina E.A., Brunet A. (2015). Epigenetic regulation of ageing: Linking environmental inputs to genomic stability. Nat. Rev. Mol. Cell Biol..

[126] Hernandez D.G., Nalls M.A., Gibbs J.R., Arepalli S., van der Brug M., Chong S., Moore M., Longo D.L., Cookson M.R., Traynor B.J. (2011). Distinct DNA methylation changes highly correlated with chronological age in the human brain. Hum. Mol. Genet..

[127] Waki T., Tamura G., Sato M., Motoyama T. (2003). Age-related methylation of tumor suppressor and tumor-related genes: An analysis of autopsy samples. Oncogene.

[128] Casillas M.A., Lopatina N., Andrews L.G., Tollefsbol T.O. (2003). Transcriptional control of the DNA methyltransferases is altered in aging and neoplastically-transformed human fibroblasts. Mol. Cell. Biochem..

[129] Li Y., Liu L., Tollefsbol T.O. (2010). Glucose restriction can extend normal cell lifespan and impair precancerous cell growth through epigenetic control of hTERT and p16 expression. FASEB J..

[130] Wakeling L.A., Ions L.J., Ford D. (2009). Could Sirt1-mediated epigenetic effects contribute to the longevity response to dietary restriction and be mimicked by other dietary interventions?. Age.

[131] Alageel A., Tomasi J., Tersigni C., Brietzke E., Zuckerman H., Subramaniapillai M., Lee Y., Iacobucci M., Rosenblat J.D., Mansur R.B. (2018). Evidence supporting a mechanistic role of sirtuins in mood and metabolic disorders. Prog. Neuropsychopharmacol. Biol. Psychiatry.

[132] De Ruijter A.J., van Gennip A.H., Caron H.N., Kemp S., van Kuilenburg A.B. (2003). Histone deacetylases (HDACs): Characterization of the classical HDAC family. Biochem. J..

[133] Satoh A., Brace C.S., Rensing N., Cliften P., Wozniak D.F., Herzog E.D., Yamada K.A., Imai S. (2013). Sirt1 extends life span and delays aging in mice through the regulation of Nk2 homeobox 1 in the DMH and LH. Cell Metab..

[134] Siebold A.P., Banerjee R., Tie F., Kiss D.L., Moskowitz J., Harte P.J. (2010). Polycomb Repressive Complex 2 and Trithorax modulate Drosophila longevity and stress resistance. Proc. Natl. Acad. Sci. USA.

[135] Pasyukova E.G., Vaiserman A.M. (2017). HDAC inhibitors: A new promising drug class in anti-aging research. Mech. Ageing Dev..

[136] Sharma S., Taliyan R. (2015). Targeting histone deacetylases: A novel approach in Parkinson’s disease. Parkinsons Dis..

[137] Yoo Y.E., Ko C.P. (2011). Treatment with trichostatin A initiated after disease onset delays disease progression and increases survival in a mouse model of amyotrophic lateral sclerosis. Exp. Neurol..

[138] Ricobaraza A., Cuadrado-Tejedor M., Marco S., Pérez-Otaño I., García-Osta A. (2012). Phenylbutyrate rescues dendritic spine loss associated with memory deficits in a mouse model of Alzheimer disease. Hippocampus.

[139] Wiley J.C., Pettan-Brewer C., Ladiges W.C. (2011). Phenylbutyric acid reduces amyloid plaques and rescues cognitive behavior in AD transgenic mice. Aging Cell.

[140] Harrison I.F., Crum W.R., Vernon A.C., Dexter D.T. (2015). Neurorestoration induced by the HDAC inhibitor sodium valproate in the lactacystin model of Parkinson’s is associated with histone acetylation and up-regulation of neurotrophic factors. Br. J. Pharmacol..

[141] Grillari J., Grillari-Voglauer R. (2010). Novel modulators of senescence, aging, and longevity: Small non-coding RNAs enter the stage. Exp. Gerontol..

[142] Nelson P.T., Wang W.X., Rajeev B.W. (2008). MicroRNAs (miRNAs) in neurodegenerative diseases. Brain Pathol..

[143] Rodriguez-Ortiz C.J., Baglietto-Vargas D., Martinez-Coria H., LaFerla F.M., Kitazawa M. (2014). Upregulation of miR-181 decreases c-Fos and SIRT-1 in the hippocampus of 3xTg-AD mice. J. Alzheimers Dis..

[144] Mao K., Zhang G. (2021). The role of PARP1 in neurodegenerative diseases and aging. FEBS J.

[145] Martire S., Mosca L., d’Erme M. (2015). PARP-1 involvement in neurodegeneration: A focus on Alzheimer’s and Parkinson’s diseases. Mech. Ageing Dev..

[146] Narne P., Pandey V., Simhadri P.K., Phanithi P.B. (2017). Poly(ADP-ribose)polymerase-1 hyperactivation in neurodegenerative diseases: The death knell tolls for neurons. Semin. Cell Dev. Biol..

[147] Beneke S., Cohausz O., Malanga M., Boukamp P., Althaus F., Bürkle A. (2008). Rapid regulation of telomere length is mediated by poly(ADP-ribose) polymerase-1. Nucleic Acids Res..

[148] Ye T.J., Lu Y.L., Yan X.F., Hu X.D., Wang X.L. (2019). High mobility group box-1 release from H_2_O_2_-injured hepatocytes due to sirt1 functional inhibition. World J. Gastroenterol..

[149] Kam T.I., Mao X., Park H., Chou S.C., Karuppagounder S.S., Umanah G.E., Yun S.P., Brahmachari S., Panicker N., Chen R. (2018). Poly(ADP-ribose) drives pathologic α-synuclein neurodegeneration in Parkinson’s disease. Science.

[150] Rulten S.L., Rotheray A., Green R.L., Grundy G.J., Moore D.A., Gómez-Herreros F., Hafezparast M., Caldecott K.W. (2014). PARP-1 dependent recruitment of the amyotrophic lateral sclerosis-associated protein FUS/TLS to sites of oxidative DNA damage. Nucleic Acids Res..

[151] Chini C.C.S., Tarragó M.G., Chini E.N. (2017). NAD and the aging process: Role in life, death and everything in between. Mol. Cell. Endocrinol..

[152] Xie Z., Zhang D., Chung D., Tang Z., Huang H., Dai L., Qi S., Li J., Colak G., Chen Y. (2016). Metabolic Regulation of Gene Expression by Histone Lysine β-Hydroxybutyrylation. Mol. Cell.

[153] Green D.R., Galluzzi L., Kroemer G. (2011). Mitochondria and the autophagy-inflammation-cell death axis in organismal aging. Science.

[154] Moehle E.A., Shen K., Dillin A. (2019). Mitochondrial proteostasis in the context of cellular and organismal health and aging. J. Biol. Chem..

[155] Hekimi S., Lapointe J., Wen Y. (2011). Taking a “good” look at free radicals in the aging process. Trends Cell Biol..

[156] Lipinski M.M., Zheng B., Lu T., Yan Z., Py B.F., Ng A., Xavier R.J., Li C., Yankner B.A., Scherzer C.R. (2010). Genome-wide analysis reveals mechanisms modulating autophagy in normal brain aging and in Alzheimer’s disease. Proc. Natl. Acad. Sci. USA.

[157] Winslow A.R., Chen C.W., Corrochano S., Acevedo-Arozena A., Gordon D.E., Peden A.A., Lichtenberg M., Menzies F.M., Ravikumar B., Imarisio S. (2010). α-Synuclein impairs macroautophagy: Implications for Parkinson’s disease. J. Cell Biol..

[158] Youle R.J., Narendra D.P. (2011). Mechanisms of mitophagy. Nat. Rev. Mol. Cell. Biol..

[159] Gottlieb R.A., Mentzer R.M. (2010). Autophagy during cardiac stress: Joys and frustrations of autophagy. Annu. Rev. Physiol..

[160] Madeo F., Tavernarakis N., Kroemer G. (2010). Can autophagy promote longevity?. Nat. Cell Biol..

[161] Zhou R., Yazdi A.S., Menu P., Tschopp J. (2011). A role for mitochondria in NLRP3 inflammasome activation. Nature.

[162] Herzig S., Shaw R.J. (2018). AMPK: Guardian of metabolism and mitochondrial homeostasis. Nat. Rev. Mol. Cell Biol..

[163] Lee I.H., Cao L., Mostoslavsky R., Lombard D.B., Liu J., Bruns N.E., Tsokos M., Alt F.W., Finkel T. (2008). A role for the NAD-dependent deacetylase Sirt1 in the regulation of autophagy. Proc. Natl. Acad. Sci. USA.

[164] McCarty M.F., DiNicolantonio J.J., O’Keefe J.H. (2015). Ketosis may promote brain macroautophagy by activating Sirt1 and hypoxia-inducible factor-1. Med. Hypotheses.

[165] Qiu X., Brown K., Hirschey M.D., Verdin E., Chen D. (2010). Calorie restriction reduces oxidative stress by SIRT3-mediated SOD2 activation. Cell Metab..

[166] Hafner A.V., Dai J., Gomes A.P., Xiao C.Y., Palmeira C.M., Rosenzweig A., Sinclair D.A. (2010). Regulation of the mPTP by SIRT3-mediated deacetylation of CypD at lysine 166 suppresses age-related cardiac hypertrophy. Aging.

[167] Halling J.F., Pilegaard H. (2020). PGC-1α-mediated regulation of mitochondrial function and physiological implications. Appl. Physiol. Nutr. Metab..

[168] Austin S., St-Pierre J. (2012). PGC1α and mitochondrial metabolism--emerging concepts and relevance in ageing and neurodegenerative disorders. J. Cell Sci..

[169] Cantó C., Auwerx J. (2009). PGC-1alpha, SIRT1 and AMPK, an energy sensing network that controls energy expenditure. Curr. Opin. Lipidol..

[170] Puigserver P., Spiegelman B.M. (2003). Peroxisome proliferator-activated receptor-gamma coactivator 1 alpha (PGC-1α): Transcriptional coactivator and metabolic regulator. Endocr. Rev..

[171] Scirpo R., Fiorotto R., Villani A., Amenduni M., Spirli C., Strazzabosco M. (2015). Stimulation of nuclear receptor peroxisome proliferator-activated receptor-γ limits NF-κB-dependent inflammation in mouse cystic fibrosis biliary epithelium. Hepatology.

[172] Tyagi S., Gupta P., Saini A.S., Kaushal C., Sharma S. (2011). The peroxisome proliferator-activated receptor: A family of nuclear receptors role in various diseases. J. Adv. Pharm. Technol. Res..

[173] Qin W., Yang T., Ho L., Zhao Z., Wang J., Chen L., Zhao W., Thiyagarajan M., MacGrogan D., Rodgers J.T. (2006). Neuronal SIRT1 activation as a novel mechanism underlying the prevention of Alzheimer disease amyloid neuropathology by calorie restriction. J. Biol. Chem..

[174] Qin W., Haroutunian V., Katsel P., Cardozo C.P., Ho L., Buxbaum J.D., Pasinetti G.M. (2009). PGC-1alpha expression decreases in the Alzheimer disease brain as a function of dementia. Arch. Neurol..

[175] Eisele P.S., Salatino S., Sobek J., Hottiger M.O., Handschin C. (2013). The peroxisome proliferator-activated receptor γ coactivator 1α/β (PGC-1) coactivators repress the transcriptional activity of NF-κB in skeletal muscle cells. J. Biol. Chem..

[176] Mookerjee S.A., Divakaruni A.S., Jastroch M., Brand M.D. (2010). Mitochondrial uncoupling and lifespan. Mech. Ageing Dev..

[177] Franceschi C., Garagnani P., Parini P., Giuliani C., Santoro A. (2018). Inflammaging: A new immune-metabolic viewpoint for age-related diseases. Nat. Rev. Endocrinol..

[178] Josephson A.M., Bradaschia-Correa V., Lee S., Leclerc K., Patel K.S., Muinos Lopez E., Litwa H.P., Neibart S.S., Kadiyala M., Wong M.Z. (2019). Age-related inflammation triggers skeletal stem/progenitor cell dysfunction. Proc. Natl. Acad. Sci. USA.

[179] Bauernfeind F., Ablasser A., Bartok E., Kim S., Schmid-Burgk J., Cavlar T., Hornung V. (2011). Inflammasomes: Current understanding and open questions. Cell. Mol. Life Sci..

[180] Salminen A., Kaarniranta K., Kauppinen A. (2012). Inflammaging: Disturbed interplay between autophagy and inflammasomes. Aging.

[181] Martinez-Vicente M., Cuervo A.M. (2007). Autophagy and neurodegeneration: When the cleaning crew goes on strike. Lancet Neurol..

[182] Nixon R.A., Yang D.S. (2011). Autophagy failure in Alzheimer’s disease--locating the primary defect. Neurobiol. Dis..

[183] Masters S.L., O’Neill L.A. (2011). Disease-associated amyloid and misfolded protein aggregates activate the inflammasome. Trends Mol. Med..

[184] Salminen A., Huuskonen J., Ojala J., Kauppinen A., Kaarniranta K., Suuronen T. (2008). Activation of innate immunity system during aging: NF-kB signaling is the molecular culprit of inflamm-aging. Ageing Res. Rev..

[185] Zhou R., Tardivel A., Thorens B., Choi I., Tschopp J. (2010). Thioredoxin-interacting protein links oxidative stress to inflammasome activation. Nat. Immunol..

[186] Levy M., Thaiss C.A., Elinav E. (2015). Taming the inflammasome. Nat. Med..

[187] Patel M.N., Carroll R.G., Galván-Peña S., Mills E.L., Olden R., Triantafilou M., Wolf A.I., Bryant C.E., Triantafilou K., Masters S.L. (2017). Inflammasome Priming in Sterile Inflammatory Disease. Trends Mol. Med..

[188] Mihaylova M.M., Shaw R.J. (2011). The AMPK signalling pathway coordinates cell growth, autophagy and metabolism. Nat. Cell Biol..

[189] Abdullah A., Mohd Murshid N., Makpol S. (2020). Antioxidant Modulation of mTOR and Sirtuin Pathways in Age-Related Neurodegenerative Diseases. Mol. Neurobiol..

[190] Osorio F.G., Bárcena C., Soria-Valles C., Ramsay A.J., de Carlos F., Cobo J., Fueyo A., Freije J.M., López-Otín C. (2012). Nuclear lamina defects cause ATM-dependent NF-κB activation and link accelerated aging to a systemic inflammatory response. Genes Dev..

[191] Amaya-Montoya M., Pérez-Londoño A., Guatibonza-García V., Vargas-Villanueva A., Mendivil C.O. (2020). Cellular Senescence as a Therapeutic Target for Age-Related Diseases: A Review. Adv. Ther..

[192] Borghesan M., Hoogaars W.M.H., Varela-Eirin M., Talma N., Demaria M. (2020). A Senescence-Centric View of Aging: Implications for Longevity and Disease. Trends Cell Biol..

[193] Campisi J. (2013). Aging, cellular senescence, and cancer. Annu. Rev. Physiol..

[194] Di Micco R., Krizhanovsky V., Baker D., d’Adda di Fagagna F. (2021). Cellular senescence in ageing: From mechanisms to therapeutic opportunities. Nat. Rev. Mol. Cell Biol..

[195] He S., Sharpless N.E. (2017). Senescence in Health and Disease. Cell.

[196] Tran D., Bergholz J., Zhang H., He H., Wang Y., Zhang Y., Li Q., Kirkland J.L., Xiao Z.X. (2014). Insulin-like growth factor-1 regulates the SIRT1-p53 pathway in cellular senescence. Aging Cell.

[197] Young A.R., Narita M., Ferreira M., Kirschner K., Sadaie M., Darot J.F., Tavaré S., Arakawa S., Shimizu S., Watt F.M. (2009). Autophagy mediates the mitotic senescence transition. Genes Dev..

[198] Chen C., Zhou M., Ge Y., Wang X. (2020). SIRT1 and aging related signaling pathways. Mech. Ageing Dev..

[199] Salmenperä P., Karhemo P.R., Räsänen K., Laakkonen P., Vaheri A. (2016). Fibroblast spheroids as a model to study sustained fibroblast quiescence and their crosstalk with tumor cells. Exp. Cell Res..

[200] Cai J., Weiss M.L., Rao M.S. (2004). In search of “stemness”. Exp. Hematol..

[201] Kim K.H., Chen C.C., Monzon R.I., Lau L.F. (2013). Matricellular protein CCN1 promotes regression of liver fibrosis through induction of cellular senescence in hepatic myofibroblasts. Mol. Cell Biol..

[202] Collado M., Serrano M. (2010). Senescence in tumours: Evidence from mice and humans. Nat. Rev. Cancer.

[203] Chinta S.J., Woods G., Demaria M., Rane A., Zou Y., McQuade A., Rajagopalan S., Limbad C., Madden D.T., Campisi J. (2018). Cellular Senescence Is Induced by the Environmental Neurotoxin Paraquat and Contributes to Neuropathology Linked to Parkinson’s Disease. Cell Rep..

[204] Finkel T., Serrano M., Blasco M.A. (2007). The common biology of cancer and ageing. Nature.

[205] Wang J.C., Bennett M. (2012). Aging and atherosclerosis: Mechanisms, functional consequences, and potential therapeutics for cellular senescence. Circ. Res..

[206] Cao L., Li W., Kim S., Brodie S.G., Deng C.X. (2003). Senescence, aging, and malignant transformation mediated by p53 in mice lacking the Brca1 full-length isoform. Genes Dev..

[207] Jones R.G., Plas D.R., Kubek S., Buzzai M., Mu J., Xu Y., Birnbaum M.J., Thompson C.B. (2005). AMP-activated protein kinase induces a p53-dependent metabolic checkpoint. Mol. Cell.

[208] Balch W.E., Morimoto R.I., Dillin A., Kelly J.W. (2008). Adapting proteostasis for disease intervention. Science.

[209] Klaips C.L., Jayaraj G.G., Hartl F.U. (2018). Pathways of cellular proteostasis in aging and disease. J. Cell Biol..

[210] Lapierre L.R., De Magalhaes Filho C.D., McQuary P.R., Chu C.C., Visvikis O., Chang J.T., Gelino S., Ong B., Davis A.E., Irazoqui J.E. (2013). The TFEB orthologue HLH-30 regulates autophagy and modulates longevity in *Caenorhabditis elegans*. Nat. Commun..

[211] Basisty N., Meyer J.G., Schilling B. (2018). Protein Turnover in Aging and Longevity. Proteomics.

[212] Wong S.Q., Kumar A.V., Mills J., Lapierre L.R. (2020). Autophagy in aging and longevity. Hum. Genet..

[213] Sorrentino V., Romani M., Mouchiroud L., Beck J.S., Zhang H., D’Amico D., Moullan N., Potenza F., Schmid A.W., Rietsch S. (2017). Enhancing mitochondrial proteostasis reduces amyloid-β proteotoxicity. Nature.

[214] Fang E.F., Hou Y., Palikaras K., Adriaanse B.A., Kerr J.S., Yang B., Lautrup S., Hasan-Olive M.M., Caponio D., Dan X. (2019). Mitophagy inhibits amyloid-β and tau pathology and reverses cognitive deficits in models of Alzheimer’s disease. Nat. Neurosci..

[215] Ryu D., Mouchiroud L., Andreux P.A., Katsyuba E., Moullan N., Nicolet-Dit-Félix A.A., Williams E.G., Jha P., Lo Sasso G., Huzard D. (2016). Urolithin A induces mitophagy and prolongs lifespan in *C. elegans* and increases muscle function in rodents. Nat. Med..

[216] Ou X., Lee M.R., Huang X., Messina-Graham S., Broxmeyer H.E. (2014). SIRT1 positively regulates autophagy and mitochondria function in embryonic stem cells under oxidative stress. Stem Cells.

[217] Goodell M.A., Rando T.A. (2015). Stem cells and healthy aging. Science.

[218] Keyes B.E., Fuchs E. (2018). Stem cells: Aging and transcriptional fingerprints. J. Cell. Biol..

[219] Zhang P., Kishimoto Y., Grammatikakis I., Gottimukkala K., Cutler R.G., Zhang S., Abdelmohsen K., Bohr V.A., Misra Sen J., Gorospe M. (2019). Senolytic therapy alleviates Aβ-associated oligodendrocyte progenitor cell senescence and cognitive deficits in an Alzheimer’s disease model. Nat. Neurosci..

[220] Ehninger D., Neff F., Xie K. (2014). Longevity, aging and rapamycin. Cell. Mol. Life Sci..

[221] Kaeberlein M., Galvan V. (2019). Rapamycin and Alzheimer’s disease: Time for a clinical trial?. Sci. Transl. Med..

[222] Sharma M., Gupta Y.K. (2002). Chronic treatment with trans resveratrol prevents intracerebroventricular streptozotocin induced cognitive impairment and oxidative stress in rats. Life Sci..

[223] Lu M., Su C., Qiao C., Bian Y., Ding J., Hu G. (2016). Metformin Prevents Dopaminergic Neuron Death in MPTP/P-Induced Mouse Model of Parkinson’s Disease via Autophagy and Mitochondrial ROS Clearance. Int. J. Neuropsychopharmacol..

[224] Foretz M., Guigas B., Bertrand L., Pollak M., Viollet B. (2014). Metformin: From mechanisms of action to therapies. Cell Metab..

[225] Kulkarni A.S., Gubbi S., Barzilai N. (2020). Benefits of Metformin in Attenuating the Hallmarks of Aging. Cell Metab..

[226] Najafi M., Cheki M., Rezapoor S., Geraily G., Motevaseli E., Carnovale C., Clementi E., Shirazi A. (2018). Metformin: Prevention of genomic instability and cancer: A review. Mutat. Res. Genet. Toxicol. Environ. Mutagen..

[227] Aatsinki S.M., Buler M., Salomäki H., Koulu M., Pavek P., Hakkola J. (2014). Metformin induces PGC-1α expression and selectively affects hepatic PGC-1α functions. Br. J. Pharmacol..

[228] Prasad S., Sajja R.K., Kaisar M.A., Park J.H., Villalba H., Liles T., Abbruscato T., Cucullo L. (2017). Role of Nrf2 and protective effects of Metformin against tobacco smoke-induced cerebrovascular toxicity. Redox Biol..

[229] Cuyàs E., Verdura S., Llorach-Parés L., Fernández-Arroyo S., Joven J., Martin-Castillo B., Bosch-Barrera J., Brunet J., Nonell-Canals A., Sanchez-Martinez M. (2018). Metformin Is a Direct SIRT1-Activating Compound: Computational Modeling and Experimental Validation. Front. Endocrinol..

[230] Moiseeva O., Deschênes-Simard X., St-Germain E., Igelmann S., Huot G., Cadar A.E., Bourdeau V., Pollak M.N., Ferbeyre G. (2013). Metformin inhibits the senescence-associated secretory phenotype by interfering with IKK/NF-κB activation. Aging Cell.

[231] Tizazu A.M., Nyunt M.S.Z., Cexus O., Suku K., Mok E., Xian C.H., Chong J., Tan C., How W., Hubert S. (2019). Metformin Monotherapy Downregulates Diabetes-Associated Inflammatory Status and Impacts on Mortality. Front. Physiol..

[232] Wang C., Liu C., Gao K., Zhao H., Zhou Z., Shen Z., Guo Y., Li Z., Yao T., Mei X. (2016). Metformin preconditioning provide neuroprotection through enhancement of autophagy and suppression of inflammation and apoptosis after spinal cord injury. Biochem. Biophys. Res. Commun..

[233] Markowicz-Piasecka M., Sikora J., Szydłowska A., Skupień A., Mikiciuk-Olasik E., Huttunen K.M. (2017). Metformin—A Future Therapy for Neurodegenerative Diseases: Theme: Drug Discovery, Development and Delivery in Alzheimer’s Disease Guest Editor: Davide Brambilla. Pharm. Res..

[234] Fang J., Yang J., Wu X., Zhang G., Li T., Wang X., Zhang H., Wang C.C., Liu G.H., Wang L. (2018). Metformin alleviates human cellular aging by upregulating the endoplasmic reticulum glutathione peroxidase 7. Aging Cell.

[235] Bridgeman S.C., Ellison G.C., Melton P.E., Newsholme P., Mamotte C.D.S. (2018). Epigenetic effects of metformin: From molecular mechanisms to clinical implications. Diabetes Obes. Metab..

[236] Noren Hooten N., Martin-Montalvo A., Dluzen D.F., Zhang Y., Bernier M., Zonderman A.B., Becker K.G., Gorospe M., de Cabo R., Evans M.K. (2016). Metformin-mediated increase in DICER1 regulates microRNA expression and cellular senescence. Aging Cell.

[237] De Zegher F., Díaz M., Ibáñez L. (2015). Association between Long Telomere Length and Insulin Sensitization in Adolescent Girls with Hyperinsulinemic Androgen Excess. JAMA Pediatr..

[238] Diman A., Boros J., Poulain F., Rodriguez J., Purnelle M., Episkopou H., Bertrand L., Francaux M., Deldicque L., Decottignies A. (2016). Nuclear respiratory factor 1 and endurance exercise promote human telomere transcription. Sci. Adv..

[239] Bhullar K.S., Hubbard B.P. (2015). Lifespan and healthspan extension by resveratrol. Biochim. Biophys. Acta.

[240] Porquet D., Casadesús G., Bayod S., Vicente A., Canudas A.M., Vilaplana J., Pelegrí C., Sanfeliu C., Camins A., Pallàs M. (2013). Dietary resveratrol prevents Alzheimer’s markers and increases life span in SAMP8. Age.

[241] Mancuso R., del Valle J., Modol L., Martinez A., Granado-Serrano A.B., Ramirez-Núñez O., Pallás M., Portero-Otin M., Osta R., Navarro X. (2014). Resveratrol improves motoneuron function and extends survival in SOD1(G93A) ALS mice. Neurotherapeutics.

[242] Price N.L., Gomes A.P., Ling A.J., Duarte F.V., Martin-Montalvo A., North B.J., Agarwal B., Ye L., Ramadori G., Teodoro J.S. (2012). SIRT1 is required for AMPK activation and the beneficial effects of resveratrol on mitochondrial function. Cell Metab..

[243] Morris B.J. (2013). Seven sirtuins for seven deadly diseases of aging. Free Radic. Biol. Med..

[244] Choi H.I., Kim H.J., Park J.S., Kim I.J., Bae E.H., Ma S.K., Kim S.W. (2017). PGC-1α attenuates hydrogen peroxide-induced apoptotic cell death by upregulating Nrf-2 via GSK3β inactivation mediated by activated p38 in HK-2 Cells. Sci. Rep..

[245] Huang K., Gao X., Wei W. (2017). The crosstalk between Sirt1 and Keap1/Nrf2/ARE anti-oxidative pathway forms a positive feedback loop to inhibit FN and TGF-β1 expressions in rat glomerular mesangial cells. Exp. Cell Res..

[246] Joo M.S., Kim W.D., Lee K.Y., Kim J.H., Koo J.H., Kim S.G. (2016). AMPK Facilitates Nuclear Accumulation of Nrf2 by Phosphorylating at Serine 550. Mol. Cell Biol..

[247] Mercken E.M., Mitchell S.J., Martin-Montalvo A., Minor R.K., Almeida M., Gomes A.P., Scheibye-Knudsen M., Palacios H.H., Licata J.J., Zhang Y. (2014). SRT2104 extends survival of male mice on a standard diet and preserves bone and muscle mass. Aging Cell.

[248] Carullo G., Cappello A.R., Frattaruolo L., Badolato M., Armentano B., Aiello F. (2017). Quercetin and derivatives: Useful tools in inflammation and pain management. Future Med. Chem..

[249] Feng X., Sureda A., Jafari S., Memariani Z., Tewari D., Annunziata G., Barrea L., Hassan S.T.S., Šmejkal K., Malaník M. (2019). Berberine in Cardiovascular and Metabolic Diseases: From Mechanisms to Therapeutics. Theranostics.

[250] Sarker M.R., Franks S.F. (2018). Efficacy of curcumin for age-associated cognitive decline: A narrative review of preclinical and clinical studies. Geroscience.

[251] Sato K., Kashiwaya Y., Keon C.A., Tsuchiya N., King M.T., Radda G.K., Chance B., Clarke K., Veech R.L. (1995). Insulin, ketone bodies, and mitochondrial energy transduction. FASEB J..

[252] Sharma A.K., Rani E., Waheed A., Rajput S.K. (2015). Pharmacoresistant Epilepsy: A Current Update on Non-Conventional Pharmacological and Non-Pharmacological Interventions. J. Epilepsy Res..

[253] VanItallie T.B., Nufert T.H. (2003). Ketones: Metabolism’s ugly duckling. Nutr. Rev..

[254] Almeida C.G., de Mendonça A., Cunha R.A., Ribeiro J.A. (2003). Adenosine promotes neuronal recovery from reactive oxygen species induced lesion in rat hippocampal slices. Neurosci. Lett..

[255] Choudhury H., Chellappan D.K., Sengupta P., Pandey M., Gorain B. (2019). Adenosine Receptors in Modulation of Central Nervous System Disorders. Curr. Pharm. Des..

[256] McNally M.A., Hartman A.L. (2012). Ketone bodies in epilepsy. J. Neurochem..

[257] Juge N., Gray J.A., Omote H., Miyaji T., Inoue T., Hara C., Uneyama H., Edwards R.H., Nicoll R.A., Moriyama Y. (2010). Metabolic control of vesicular glutamate transport and release. Neuron.

[258] Tieu K., Perier C., Caspersen C., Teismann P., Wu D.C., Yan S.D., Naini A., Vila M., Jackson-Lewis V., Ramasamy R. (2003). D-beta-hydroxybutyrate rescues mitochondrial respiration and mitigates features of Parkinson disease. J. Clin. Investig..

[259] Veech R.L., Todd King M., Pawlosky R., Kashiwaya Y., Bradshaw P.C., Curtis W. (2019). The “great” controlling nucleotide coenzymes. IUBMB Life.

[260] Shimazu T., Hirschey M.D., Newman J., He W., Shirakawa K., Le Moan N., Grueter C.A., Lim H., Saunders L.R., Stevens R.D. (2013). Suppression of oxidative stress by β-hydroxybutyrate, an endogenous histone deacetylase inhibitor. Science.

[261] Tseng A.H., Shieh S.S., Wang D.L. (2013). SIRT3 deacetylates FOXO3 to protect mitochondria against oxidative damage. Free Radic. Biol. Med..

[262] Zhao L., Ackerman S.L. (2006). Endoplasmic reticulum stress in health and disease. Curr. Opin. Cell Biol..

[263] Sleiman S.F., Henry J., Al-Haddad R., El Hayek L., Abou Haidar E., Stringer T., Ulja D., Karuppagounder S.S., Holson E.B., Ratan R.R. (2016). Exercise promotes the expression of brain derived neurotrophic factor (BDNF) through the action of the ketone body β-hydroxybutyrate. Elife.

[264] Manning B.D., Cantley L.C. (2007). AKT/PKB signaling: Navigating downstream. Cell.

[265] Xu D., Lian D., Wu J., Liu Y., Zhu M., Sun J., He D., Li L. (2017). Brain-derived neurotrophic factor reduces inflammation and hippocampal apoptosis in experimental Streptococcus pneumoniae meningitis. J. Neuroinflamm..

[266] Marosi K., Kim S.W., Moehl K., Scheibye-Knudsen M., Cheng A., Cutler R., Camandola S., Mattson M.P. (2016). 3-Hydroxybutyrate regulates energy metabolism and induces BDNF expression in cerebral cortical neurons. J. Neurochem..

[267] Lau D., Bengtson C.P., Buchthal B., Bading H. (2015). BDNF Reduces Toxic Extrasynaptic NMDA Receptor Signaling via Synaptic NMDA Receptors and Nuclear-Calcium-Induced Transcription of inhba/Activin A. Cell Rep..

[268] Mattson M.P., Lovell M.A., Furukawa K., Markesbery W.R. (1995). Neurotrophic factors attenuate glutamate-induced accumulation of peroxides, elevation of intracellular Ca^2+^ concentration, and neurotoxicity and increase antioxidant enzyme activities in hippocampal neurons. J. Neurochem..

[269] Menzies K.J., Zhang H., Katsyuba E., Auwerx J. (2016). Protein acetylation in metabolism—Metabolites and cofactors. Nat. Rev. Endocrinol..

[270] Guil S., Long J.C., Cáceres J.F. (2006). hnRNP A1 relocalization to the stress granules reflects a role in the stress response. Mol. Cell. Biol..

[271] Jean-Philippe J., Paz S., Caputi M. (2013). hnRNP A1: The Swiss army knife of gene expression. Int. J. Mol. Sci..

[272] Parodi B., Rossi S., Morando S., Cordano C., Bragoni A., Motta C., Usai C., Wipke B.T., Scannevin R.H., Mancardi G.L. (2015). Fumarates modulate microglia activation through a novel HCAR2 signaling pathway and rescue synaptic dysregulation in inflamed CNS. Acta Neuropathol..

[273] Maalouf M., Sullivan P.G., Davis L., Kim D.Y., Rho J.M. (2007). Ketones inhibit mitochondrial production of reactive oxygen species production following glutamate excitotoxicity by increasing NADH oxidation. Neuroscience.

[274] Pawlosky R.J., Kemper M.F., Kashiwaya Y., King M.T., Mattson M.P., Veech R.L. (2017). Effects of a dietary ketone ester on hippocampal glycolytic and tricarboxylic acid cycle intermediates and amino acids in a 3xTgAD mouse model of Alzheimer’s disease. J. Neurochem..

[275] Offermanns S., Schwaninger M. (2015). Nutritional or pharmacological activation of HCA(2) ameliorates neuroinflammation. Trends Mol. Med..

[276] Shabab T., Khanabdali R., Moghadamtousi S.Z., Kadir H.A., Mohan G. (2017). Neuroinflammation pathways: A general review. Int. J. Neurosci..

[277] Norwitz N.G., Hu M.T., Clarke K. (2019). The Mechanisms by Which the Ketone Body D-β-Hydroxybutyrate May Improve the Multiple Cellular Pathologies of Parkinson’s disease. Front. Nutr..

[278] Hasan-Olive M.M., Lauritzen K.H., Ali M., Rasmussen L.J., Storm-Mathisen J., Bergersen L.H. (2019). A Ketogenic Diet Improves Mitochondrial Biogenesis and Bioenergetics via the PGC1α-SIRT3-UCP2 Axis. Neurochem. Res..

[279] Zou X.H., Li H.M., Wang S., Leski M., Yao Y.C., Yang X.D., Huang Q.J., Chen G.Q. (2009). The effect of 3-hydroxybutyrate methyl ester on learning and memory in mice. Biomaterials.

[280] Scheibye-Knudsen M., Mitchell S.J., Fang E.F., Iyama T., Ward T., Wang J., Dunn C.A., Singh N., Veith S., Hasan-Olive M.M. (2014). A high-fat diet and NAD+ activate Sirt1 to rescue premature aging in cockayne syndrome. Cell Metab..

[281] Houtkooper R.H., Auwerx J. (2012). Exploring the therapeutic space around NAD+. J. Cell Biol..

[282] Kong X., Wang R., Xue Y., Liu X., Zhang H., Chen Y., Fang F., Chang Y. (2010). Sirtuin 3, a new target of PGC-1alpha, plays an important role in the suppression of ROS and mitochondrial biogenesis. PLoS ONE.

[283] Shi T., Wang F., Stieren E., Tong Q. (2005). SIRT3, a mitochondrial sirtuin deacetylase, regulates mitochondrial function and thermogenesis in brown adipocytes. J. Biol. Chem..

[284] Cheng A., Wan R., Yang J.L., Kamimura N., Son T.G., Ouyang X., Luo Y., Okun E., Mattson M.P. (2012). Involvement of PGC-1α in the formation and maintenance of neuronal dendritic spines. Nat. Commun..

[285] Jeong E.A., Jeon B.T., Shin H.J., Kim N., Lee D.H., Kim H.J., Kang S.S., Cho G.J., Choi W.S., Roh G.S. (2011). Ketogenic diet-induced peroxisome proliferator-activated receptor-γ activation decreases neuroinflammation in the mouse hippocampus after kainic acid-induced seizures. Exp. Neurol..

[286] Morris G., Puri B.K., Carvalho A., Maes M., Berk M., Ruusunen A., Olive L. (2020). Induced Ketosis as a Treatment for Neuroprogressive Disorders: Food for Thought?. Int. J. Neuropsychopharmacol..

[287] Simeone T.A., Matthews S.A., Samson K.K., Simeone K.A. (2017). Regulation of brain PPARgamma2 contributes to ketogenic diet anti-seizure efficacy. Exp. Neurol..

[288] Kashiwaya Y., Pawlosky R., Markis W., King M.T., Bergman C., Srivastava S., Murray A., Clarke K., Veech R.L. (2010). A ketone ester diet increases brain malonyl-CoA and Uncoupling proteins 4 and 5 while decreasing food intake in the normal Wistar Rat. J. Biol. Chem..

[289] Srivastava S., Kashiwaya Y., King M.T., Baxa U., Tam J., Niu G., Chen X., Clarke K., Veech R.L. (2012). Mitochondrial biogenesis and increased uncoupling protein 1 in brown adipose tissue of mice fed a ketone ester diet. FASEB J..

[290] Bae H.R., Kim D.H., Park M.H., Lee B., Kim M.J., Lee E.K., Chung K.W., Kim S.M., Im D.S., Chung H.Y. (2016). β-Hydroxybutyrate suppresses inflammasome formation by ameliorating endoplasmic reticulum stress via AMPK activation. Oncotarget.

[291] Yamanashi T., Iwata M., Kamiya N., Tsunetomi K., Kajitani N., Wada N., Iitsuka T., Yamauchi T., Miura A., Pu S. (2017). Beta-hydroxybutyrate, an endogenic NLRP3 inflammasome inhibitor, attenuates stress-induced behavioral and inflammatory responses. Sci. Rep..

[292] Youm Y.H., Nguyen K.Y., Grant R.W., Goldberg E.L., Bodogai M., Kim D., D’Agostino D., Planavsky N., Lupfer C., Kanneganti T.D. (2015). The ketone metabolite β-hydroxybutyrate blocks NLRP3 inflammasome-mediated inflammatory disease. Nat. Med..

[293] Ari C., Murdun C., Koutnik A.P., Goldhagen C.R., Rogers C., Park C., Bharwani S., Diamond D.M., Kindy M.S., D’Agostino D.P. (2019). Exogenous Ketones Lower Blood Glucose Level in Rested and Exercised Rodent Models. Nutrients.

[294] Edwards C., Copes N., Bradshaw P.C. (2015). D-ß-hydroxybutyrate: An anti-aging ketone body. Oncotarget.

[295] Kenyon C. (2011). The first long-lived mutants: Discovery of the insulin/IGF-1 pathway for ageing. Philos. Trans. R. Soc. Lond. B. Biol. Sci..

[296] Willcox B.J., Donlon T.A., He Q., Chen R., Grove J.S., Yano K., Masaki K.H., Willcox D.C., Rodriguez B., Curb J.D. (2008). FOXO3A genotype is strongly associated with human longevity. Proc. Natl. Acad. Sci. USA.

[297] Hansen M., Chandra A., Mitic L.L., Onken B., Driscoll M., Kenyon C. (2008). A role for autophagy in the extension of lifespan by dietary restriction in *C. elegans*. PLoS Genet..

[298] Yamada T., Zhang S.J., Westerblad H., Katz A. (2010). β-Hydroxybutyrate inhibits insulin-mediated glucose transport in mouse oxidative muscle. Am. J. Physiol. Endocrinol. Metab..

[299] Barja G. (2004). Free radicals and aging. Trends Neurosci..

[300] Newman J.C., Covarrubias A.J., Zhao M., Yu X., Gut P., Ng C.P., Huang Y., Haldar S., Verdin E. (2017). Ketogenic Diet Reduces Midlife Mortality and Improves Memory in Aging Mice. Cell Metab..

[301] Simeone K.A., Matthews S.A., Rho J.M., Simeone T.A. (2016). Ketogenic diet treatment increases longevity in Kcna1-null mice, a model of sudden unexpected death in epilepsy. Epilepsia.

[302] Liu K., Li F., Sun Q., Lin N., Han H., You K., Tian F., Mao Z., Li T., Tong T. (2019). p53 β-hydroxybutyrylation attenuates p53 activity. Cell Death Dis..

[303] Habieb M.E., Mohamed M.A., El Gamal D.M., Hawas A.M., Mohamed T.M. (2021). Anti-aging effect of DL-β-hydroxybutyrate against hepatic cellular senescence induced by D-galactose or γ-irradiation via autophagic flux stimulation in male rats. Arch. Gerontol. Geriatr..

[304] Wang H., Han L., Zhao G., Shen H., Wang P., Sun Z., Xu C., Su Y., Li G., Tong T. (2016). hnRNP A1 antagonizes cellular senescence and senescence-associated secretory phenotype via regulation of SIRT1 mRNA stability. Aging Cell.

[305] Armada-Moreira A., Gomes J.I., Pina C.C., Savchak O.K., Gonçalves-Ribeiro J., Rei N., Pinto S., Morais T.P., Martins R.S., Ribeiro F.F. (2020). Going the Extra (Synaptic) Mile: Excitotoxicity as the Road Toward Neurodegenerative Diseases. Front. Cell. Neurosci..

[306] Coyle J.T., Puttfarcken P. (1993). Oxidative stress, glutamate, and neurodegenerative disorders. Science.

[307] Holper L., Ben-Shachar D., Mann J.J. (2019). Multivariate meta-analyses of mitochondrial complex I and IV in major depressive disorder, bipolar disorder, schizophrenia, Alzheimer disease, and Parkinson disease. Neuropsychopharmacology.

[308] Mosconi L., de Leon M., Murray J., Lu J., Javier E., McHugh P., Swerdlow R.H. (2011). Reduced mitochondria cytochrome oxidase activity in adult children of mothers with Alzheimer’s disease. J. Alzheimers Dis..

[309] Lugrin J., Rosenblatt-Velin N., Parapanov R., Liaudet L. (2014). The role of oxidative stress during inflammatory processes. Biol. Chem..

[310] Wu Z., Yu J., Zhu A., Nakanishi H. (2016). Nutrients, Microglia Aging, and Brain Aging. Oxid. Med. Cell Longev..

[311] Hatanpää K., Isaacs K.R., Shirao T., Brady D.R., Rapoport S.I. (1999). Loss of proteins regulating synaptic plasticity in normal aging of the human brain and in Alzheimer disease. J. Neuropathol. Exp. Neurol..

[312] Rao J.S., Kellom M., Kim H.W., Rapoport S.I., Reese E.A. (2012). Neuroinflammation and synaptic loss. Neurochem. Res..

[313] Scheff S.W., Price D.A., Schmitt F.A., Mufson E.J. (2006). Hippocampal synaptic loss in early Alzheimer’s disease and mild cognitive impairment. Neurobiol. Aging.

[314] Appelberg K.S., Hovda D.A., Prins M.L. (2009). The effects of a ketogenic diet on behavioral outcome after controlled cortical impact injury in the juvenile and adult rat. J. Neurotrauma.

[315] Krikorian R., Shidler M.D., Dangelo K., Couch S.C., Benoit S.C., Clegg D.J. (2012). Dietary ketosis enhances memory in mild cognitive impairment. Neurobiol. Aging.

[316] Maalouf M., Rho J.M. (2008). Oxidative impairment of hippocampal long-term potentiation involves activation of protein phosphatase 2A and is prevented by ketone bodies. J. Neurosci. Res..

[317] Rusek M., Pluta R., Ułamek-Kozioł M., Czuczwar S.J. (2019). Ketogenic Diet in Alzheimer’s disease. Int. J. Mol. Sci..

[318] Chatterjee P., Fernando M., Fernando B., Dias C.B., Shah T., Silva R., Williams S., Pedrini S., Hillebrandt H., Goozee K. (2020). Potential of coconut oil and medium chain triglycerides in the prevention and treatment of Alzheimer’s disease. Mech. Ageing Dev..

[319] Fernando W.M., Martins I.J., Goozee K.G., Brennan C.S., Jayasena V., Martins R.N. (2015). The role of dietary coconut for the prevention and treatment of Alzheimer’s disease: Potential mechanisms of action. Br. J. Nutr..

[320] Rebello C.J., Keller J.N., Liu A.G., Johnson W.D., Greenway F.L. (2015). Pilot feasibility and safety study examining the effect of medium chain triglyceride supplementation in subjects with mild cognitive impairment: A randomized controlled trial. BBA Clin..

[321] Ota M., Matsuo J., Ishida I., Hattori K., Teraishi T., Tonouchi H., Ashida K., Takahashi T., Kunugi H. (2016). Effect of a ketogenic meal on cognitive function in elderly adults: Potential for cognitive enhancement. Psychopharmacology.

[322] Pan Y., Larson B., Araujo J.A., Lau W., de Rivera C., Santana R., Gore A., Milgram N.W. (2010). Dietary supplementation with medium-chain TAG has long-lasting cognition-enhancing effects in aged dogs. Br. J. Nutr..

[323] Pawlosky R.J., Kashiwaya Y., King M.T., Veech R.L. (2020). A Dietary Ketone Ester Normalizes Abnormal Behavior in a Mouse Model of Alzheimer’s Disease. Int. J. Mol. Sci..

[324] Alexander G.E., Chen K., Pietrini P., Rapoport S.I., Reiman E.M. (2002). Longitudinal PET Evaluation of Cerebral Metabolic Decline in Dementia: A Potential Outcome Measure in Alzheimer’s Disease Treatment Studies. Am. J. Psychiatry.

[325] Reiman E.M., Chen K., Alexander G.E., Caselli R.J., Bandy D., Osborne D., Saunders A.M., Hardy J. (2004). Functional brain abnormalities in young adults at genetic risk for late-onset Alzheimer’s dementia. Proc. Natl. Acad. Sci. USA.

[326] Henderson S.T., Vogel J.L., Barr L.J., Garvin F., Jones J.J., Costantini L.C. (2009). Study of the ketogenic agent AC-1202 in mild to moderate Alzheimer’s disease: A randomized, double-blind, placebo-controlled, multicenter trial. Nutr. Metab..

[327] Reger M.A., Henderson S.T., Hale C., Cholerton B., Baker L.D., Watson G.S., Hyde K., Chapman D., Craft S. (2004). Effects of beta-hydroxybutyrate on cognition in memory-impaired adults. Neurobiol. Aging.

[328] Kashiwaya Y., Takeshima T., Mori N., Nakashima K., Clarke K., Veech R.L. (2000). D-beta-hydroxybutyrate protects neurons in models of Alzheimer’s and Parkinson’s disease. Proc. Natl. Acad. Sci. USA.

[329] Yin J.X., Maalouf M., Han P., Zhao M., Gao M., Dharshaun T., Ryan C., Whitelegge J., Wu J., Eisenberg D. (2016). Ketones block amyloid entry and improve cognition in an Alzheimer’s model. Neurobiol. Aging.

[330] Singer T.P., Ramsay R.R., McKeown K., Trevor A., Castagnoli N.E. (1988). Mechanism of the neurotoxicity of 1-methyl-4-phenylpyridinium (MPP+), the toxic bioactivation product of 1-methyl-4-phenyl-1,2,3,6-tetrahydropyridine (MPTP). Toxicology.

[331] Vanitallie T.B., Nonas C., Di Rocco A., Boyar K., Hyams K., Heymsfield S.B. (2005). Treatment of Parkinson disease with diet-induced hyperketonemia: A feasibility study. Neurology.

[332] Ari C., Poff A.M., Held H.E., Landon C.S., Goldhagen C.R., Mavromates N., D’Agostino D.P. (2014). Metabolic therapy with Deanna Protocol supplementation delays disease progression and extends survival in amyotrophic lateral sclerosis (ALS) mouse model. PLoS ONE.

[333] Ari C., Murdun C., Goldhagen C., Koutnik A.P., Bharwani S.R., Diamond D.M., Kindy M., D’Agostino D.P., Kovacs Z. (2020). Exogenous Ketone Supplements Improved Motor Performance in Preclinical Rodent Models. Nutrients.

[334] Netzahualcoyotzi C., Tapia R. (2015). Degeneration of spinal motor neurons by chronic AMPA-induced excitotoxicity in vivo and protection by energy substrates. Acta Neuropathol. Commun..

[335] Zhao Z., Lange D.J., Voustianiouk A., MacGrogan D., Ho L., Suh J., Humala N., Thiyagarajan M., Wang J., Pasinetti G.M. (2006). A ketogenic diet as a potential novel therapeutic intervention in amyotrophic lateral sclerosis. BMC Neurosci..

[336] Zhao W., Varghese M., Vempati P., Dzhun A., Cheng A., Wang J., Lange D., Bilski A., Faravelli I., Pasinetti G.M. (2012). Caprylic triglyceride as a novel therapeutic approach to effectively improve the performance and attenuate the symptoms due to the motor neuron loss in ALS disease. PLoS ONE.

[337] Abg Abd Wahab D.Y., Gau C.H., Zakaria R., Muthu Karuppan M.K., A-Rahbi B.S., Abdullah Z., Alrafiah A., Abdullah J.M., Muthuraju S. (2019). Review on Cross Talk between Neurotransmitters and Neuroinflammation in Striatum and Cerebellum in the Mediation of Motor Behaviour. Biomed. Res. Int..

[338] Blasco H., Mavel S., Corcia P., Gordon P.H. (2014). The glutamate hypothesis in ALS: Pathophysiology and drug development. Curr. Med. Chem..

[339] Brichta L., Greengard P., Flajolet M. (2013). Advances in the pharmacological treatment of Parkinson’s disease: Targeting neurotransmitter systems. Trends Neurosci..

[340] Tisch S., Silberstein P., Limousin-Dowsey P., Jahanshahi M. (2004). The basal ganglia: Anatomy, physiology, and pharmacology. Psychiatr. Clin..

[341] D’Amelio M., Puglisi-Allegra S., Mercuri N. (2018). The role of dopaminergic midbrain in Alzheimer’s disease: Translating basic science into clinical practice. Pharmacol. Res..

[342] Huang D., Liu D., Yin J., Qian T., Shrestha S., Ni H. (2017). Glutamate-glutamine and GABA in brain of normal aged and patients with cognitive impairment. Eur. Radiol..

[343] Stanciu G.D., Luca A., Rusu R.N., Bild V., Beschea Chiriac S.I., Solcan C., Bild W., Ababei D.C. (2019). Alzheimer’s Disease Pharmacotherapy in Relation to Cholinergic System Involvement. Biomolecules.

[344] Ma S., Hangya B., Leonard C.S., Wisden W., Gundlach A.L. (2018). Dual-transmitter systems regulating arousal, attention, learning and memory. Neurosci. Biobehav. Rev..

[345] Choong C.J., Sasaki T., Hayakawa H., Yasuda T., Baba K., Hirata Y., Uesato S., Mochizuki H. (2016). A novel histone deacetylase 1 and 2 isoform-specific inhibitor alleviates experimental Parkinson’s disease. Neurobiol. Aging.

[346] D’Mello S.R. (2009). Histone deacetylases as targets for the treatment of human neurodegenerative diseases. Drug News Perspect..

[347] Feng H.L., Leng Y., Ma C.H., Zhang J., Ren M., Chuang D.M. (2008). Combined lithium and valproate treatment delays disease onset, reduces neurological deficits and prolongs survival in an amyotrophic lateral sclerosis mouse model. Neuroscience.

[348] Lu X., Wang L., Yu C., Yu D., Yu G. (2015). Histone Acetylation Modifiers in the Pathogenesis of Alzheimer’s Disease. Front. Cell. Neurosci..

[349] Chuang D.M., Leng Y., Marinova Z., Kim H.J., Chiu C.T. (2009). Multiple roles of HDAC inhibition in neurodegenerative conditions. Trends Neurosci..

[350] Kazantsev A.G., Thompson L.M. (2008). Therapeutic application of histone deacetylase inhibitors for central nervous system disorders. Nat. Rev. Drug Discov..

[351] Peleg S., Sananbenesi F., Zovoilis A., Burkhardt S., Bahari-Javan S., Agis-Balboa R.C., Cota P., Wittnam J.L., Gogol-Doering A., Opitz L. (2010). Altered histone acetylation is associated with age-dependent memory impairment in mice. Science.

[352] Sharma S., Taliyan R., Ramagiri S. (2015). Histone deacetylase inhibitor, trichostatin A, improves learning and memory in high-fat diet-induced cognitive deficits in mice. J. Mol. Neurosci..

[353] Peng S., Wuu J., Mufson E.J., Fahnestock M. (2005). Precursor form of brain-derived neurotrophic factor and mature brain-derived neurotrophic factor are decreased in the pre-clinical stages of Alzheimer’s disease. J. Neurochem..

[354] Qin X.Y., Cao C., Cawley N.X., Liu T.T., Yuan J., Loh Y.P., Cheng Y. (2017). Decreased peripheral brain-derived neurotrophic factor levels in Alzheimer’s disease: A meta-analysis study (N = 7277). Mol. Psychiatry.

[355] Valenzuela P.L., Castillo-García A., Morales J.S., de la Villa P., Hampel H., Emanuele E., Lista S., Lucia A. (2020). Exercise benefits on Alzheimer’s disease: State-of-the-science. Ageing Res. Rev..

[356] Glass C.K., Saijo K., Winner B., Marchetto M.C., Gage F.H. (2010). Mechanisms underlying inflammation in neurodegeneration. Cell.

[357] Swanton T., Cook J., Beswick J.A., Freeman S., Lawrence C.B., Brough D. (2018). Is Targeting the Inflammasome a Way Forward for Neuroscience Drug Discovery?. SLAS Discov..

[358] Hyun D.H., Lee M., Halliwell B., Jenner P. (2003). Proteasomal inhibition causes the formation of protein aggregates containing a wide range of proteins, including nitrated proteins. J. Neurochem..

[359] Lim J.E., Song M., Jin J., Kou J., Pattanayak A., Lalonde R., Fukuchi K. (2012). The effects of MyD88 deficiency on exploratory activity, anxiety, motor coordination, and spatial learning in C57BL/6 and APPswe/PS1dE9 mice. Behav. Brain Res..

[360] Liu X., Wu Z., Hayashi Y., Nakanishi H. (2012). Age-dependent neuroinflammatory responses and deficits in long-term potentiation in the hippocampus during systemic inflammation. Neuroscience.

[361] Kim D.Y., Hao J., Liu R., Turner G., Shi F.D., Rho J.M. (2012). Inflammation-mediated memory dysfunction and effects of a ketogenic diet in a murine model of multiple sclerosis. PLoS ONE.

[362] Pallàs M., Pizarro J.G., Gutierrez-Cuesta J., Crespo-Biel N., Alvira D., Tajes M., Yeste-Velasco M., Folch J., Canudas A.M., Sureda F.X. (2008). Modulation of SIRT1 expression in different neurodegenerative models and human pathologies. Neuroscience.

[363] Herskovits A.Z., Guarente L. (2014). SIRT1 in neurodevelopment and brain senescence. Neuron.

[364] Corpas R., Revilla S., Ursulet S., Castro-Freire M., Kaliman P., Petegnief V., Giménez-Llort L., Sarkis C., Pallàs M., Sanfeliu C. (2017). SIRT1 Overexpression in Mouse Hippocampus Induces Cognitive Enhancement through Proteostatic and Neurotrophic Mechanisms. Mol. Neurobiol..

[365] Wang R., Zhang Y., Li J., Zhang C. (2017). Resveratrol ameliorates spatial learning memory impairment induced by Aβ_1-42_ in rats. Neuroscience.

[366] Gao J., Wang W.Y., Mao Y.W., Gräff J., Guan J.S., Pan L., Mak G., Kim D., Su S.C., Tsai L.H. (2010). A novel pathway regulates memory and plasticity via SIRT1 and miR-134. Nature.

[367] Michán S., Li Y., Chou M.M., Parrella E., Ge H., Long J.M., Allard J.S., Lewis K., Miller M., Xu W. (2010). SIRT1 is essential for normal cognitive function and synaptic plasticity. J. Neurosci..

[368] Han S., Choi J.R., Soon Shin K., Kang S.J. (2012). Resveratrol upregulated heat shock proteins and extended the survival of G93A-SOD1 mice. Brain Res..

[369] Wang J., Zhang Y., Tang L., Zhang N., Fan D. (2011). Protective effects of resveratrol through the up-regulation of SIRT1 expression in the mutant hSOD1-G93A-bearing motor neuron-like cell culture model of amyotrophic lateral sclerosis. Neurosci. Lett..

[370] Mudò G., Mäkelä J., Di Liberto V., Tselykh T.V., Olivieri M., Piepponen P., Eriksson O., Mälkiä A., Bonomo A., Kairisalo M. (2012). Transgenic expression and activation of PGC-1α protect dopaminergic neurons in the MPTP mouse model of Parkinson’s disease. Cell. Mol. Life Sci..

[371] Karuppagounder S.S., Pinto J.T., Xu H., Chen H.L., Beal M.F., Gibson G.E. (2009). Dietary supplementation with resveratrol reduces plaque pathology in a transgenic model of Alzheimer’s disease. Neurochem. Int..

[372] St-Pierre J., Drori S., Uldry M., Silvaggi J.M., Rhee J., Jäger S., Handschin C., Zheng K., Lin J., Yang W. (2006). Suppression of reactive oxygen species and neurodegeneration by the PGC-1 transcriptional coactivators. Cell.

[373] Lin J., Wu P.H., Tarr P.T., Lindenberg K.S., St-Pierre J., Zhang C.Y., Mootha V.K., Jäger S., Vianna C.R., Reznick R.M. (2004). Defects in adaptive energy metabolism with CNS-linked hyperactivity in PGC-1alpha null mice. Cell.

[374] Wareski P., Vaarmann A., Choubey V., Safiulina D., Liiv J., Kuum M., Kaasik A. (2009). PGC-1{alpha} and PGC-1{beta} regulate mitochondrial density in neurons. J. Biol. Chem..

[375] Zheng B., Liao Z., Locascio J.J., Lesniak K.A., Roderick S.S., Watt M.L., Eklund A.C., Zhang-James Y., Kim P.D., Hauser M.A. (2010). PGC-1α, a potential therapeutic target for early intervention in Parkinson’s disease. Sci. Transl. Med..

[376] Gong B., Chen F., Pan Y., Arrieta-Cruz I., Yoshida Y., Haroutunian V., Pasinetti G.M. (2010). SCFFbx2-E3-ligase-mediated degradation of BACE1 attenuates Alzheimer’s disease amyloidosis and improves synaptic function. Aging Cell.

[377] Kiaei M., Kipiani K., Chen J., Calingasan N.Y., Beal M.F. (2005). Peroxisome proliferator-activated receptor-gamma agonist extends survival in transgenic mouse model of amyotrophic lateral sclerosis. Exp. Neurol..

[378] Zhao W., Varghese M., Yemul S., Pan Y., Cheng A., Marano P., Hassan S., Vempati P., Chen F., Qian X. (2011). Peroxisome proliferator activator receptor gamma coactivator-1alpha (PGC-1α) improves motor performance and survival in a mouse model of amyotrophic lateral sclerosis. Mol. Neurodegener..

[379] Agarwal S., Yadav A., Chaturvedi R.K. (2017). Peroxisome proliferator-activated receptors (PPARs) as therapeutic target in neurodegenerative disorders. Biochem. Biophys. Res. Commun..

[380] D’ANGELO M., Castelli V., Catanesi M., Antonosante A., Dominguez-Benot R., Ippoliti R., Benedetti E., Cimini A. (2019). PPARγ and Cognitive Performance. Int. J. Mol. Sci..

[381] Im J.Y., Lee K.W., Woo J.M., Junn E., Mouradian M.M. (2012). DJ-1 induces thioredoxin 1 expression through the Nrf2 pathway. Hum. Mol. Genet..

[382] Kensler T.W., Wakabayashi N., Biswal S. (2007). Cell survival responses to environmental stresses via the Keap1-Nrf2-ARE pathway. Annu. Rev. Pharmacol. Toxicol..

[383] Kincaid B., Bossy-Wetzel E. (2013). Forever young: SIRT3 a shield against mitochondrial meltdown, aging, and neurodegeneration. Front. Aging Neurosci..

[384] Ramesh S., Govindarajulu M., Lynd T., Briggs G., Adamek D., Jones E., Heiner J., Majrashi M., Moore T., Amin R. (2018). SIRT3 activator Honokiol attenuates β-Amyloid by modulating amyloidogenic pathway. PLoS ONE.

[385] Song W., Song Y., Kincaid B., Bossy B., Bossy-Wetzel E. (2013). Mutant SOD1G93A triggers mitochondrial fragmentation in spinal cord motor neurons: Neuroprotection by SIRT3 and PGC-1α. Neurobiol. Dis..

